# Tumour necrosis factor alpha, interleukin 1 beta and interferon gamma have detrimental effects on equine tenocytes that cannot be rescued by IL-1RA or mesenchymal stromal cell–derived factors

**DOI:** 10.1007/s00441-022-03726-6

**Published:** 2022-12-22

**Authors:** Emily J. Smith, Ross E. Beaumont, Alyce McClellan, Cheryl Sze, Esther Palomino Lago, Liberty Hazelgrove, Jayesh Dudhia, Roger K. W. Smith, Deborah J. Guest

**Affiliations:** 1 Department of Clinical Sciences and Services, The Royal Veterinary College, Hawkshead Lane, North Mymms, Hatfield, Herts AL9 7TA UK; 2 Centre for Preventative Medicine, Animal Health Trust, Newmarket, Suffolk, CB8 7UU UK; 3 Kingston University, River House, 53-57 High Street, Kingston upon Thames, Surrey, KT1 1LQ UK

**Keywords:** Inflammation, Cytokine, Tendon, Horse, Mesenchymal stem/stromal cell

## Abstract

Tendon injuries occur commonly in both human and equine athletes, and poor tendon regeneration leads to functionally deficient scar tissue and an increased frequency of re-injury. Despite evidence suggesting inadequate resolution of inflammation leads to fibrotic healing, our understanding of the inflammatory pathways implicated in tendinopathy remains poorly understood, meaning successful targeted treatments are lacking. Here, we demonstrate IL-1β, TNFα and IFN-γ work synergistically to induce greater detrimental consequences for equine tenocytes than when used individually. This includes altering tendon associated and matrix metalloproteinase gene expression and impairing the cells’ ability to contract a 3-D collagen gel, a culture technique which more closely resembles the in vivo environment. Moreover, these adverse effects cannot be rescued by direct suppression of IL-1β using IL-1RA or factors produced by BM-MSCs. Furthermore, we provide evidence that NF-κB, but not JNK, P38 MAPK or STAT 1, is translocated to the nucleus and able to bind to DNA in tenocytes following TNFα and IL-1β stimulation, suggesting this signalling cascade may be responsible for the adverse downstream consequences of these inflammatory cytokines. We suggest a superior approach for treatment of tendinopathy may therefore be to target specific signalling pathways such as NF-κB.

**Supplementary Information:**

The online version contains supplementary material available at 10.1007/s00441-022-03726-6.

## Introduction

Tendon injuries are a major cause of musculoskeletal morbidity in equine athletes. In racing Thoroughbreds, tendon injuries account for 46% of all limb injuries on racecourses, with the superficial digital flexor tendon (SDFT) being most frequently affected (Williams et al. 2001). The comparable nature of the equine SDFT and human Achilles tendon, both acting as an elastic energy store for high-speed locomotion, makes the horse an attractive model for the study of equivalent human injury (Patterson-Kane et al. 2012; Sobhani et al. 2013; Cassel et al. 2015; Ellis et al. 2022). Post-injury, the healing response is inadequate leading to the formation of inferior quality scar-like tissue. This compromises the tendons structural integrity leading to prolonged recouperation periods, increased retirement rate and high re-injury rates of up to 67% in equine athletes (Dyson 2004; Dakin et al. 2012). Although tendon injuries pose an important clinical problem, our complete understanding of the mechanisms underpinning dysregulated healing is lacking.

Historically, tendinopathy was described as a degenerative condition devoid of inflammation, as inflammatory cells were rarely identified in histological studies (Alfredson and Lorentzon 2002). However, modern molecular techniques have identified a strong inflammatory component to acute tendinopathy, with upregulation of pro-inflammatory cytokines including IFN-γ (interferon gamma), TNFα (tumour necrosis factor alpha), IL-1β (interleukin 1 beta) and IL-6 (interleukin 6) within the tendon matrix microenvironment (Hosaka et al. 2002; Dakin et al. 2012; Dakin et al. 2014; Millar et al. 2017; Morita et al. 2017). Although moderate levels of inflammation are required to initiate tendon repair, growing evidence suggests inadequate resolution of inflammation contributes to fibrotic healing (Dakin et al. 2012; Abraham et al. 2017; Millar et al. 2017). Previously, in vitro studies have demonstrated that TNFα and IL-1β used individually exhibit detrimental consequences for equine adult tenocyte function by significantly increasing the expression of matrix metalloproteinases (MMPs) and altering the expression of tendon-associated genes (John et al. 2010; McClellan et al. 2019a, b). Additionally, IL-1β has been shown to impair three-dimensional (3-D) collagen gel contraction by tenocytes (McClellan et al. 2019a), a culture technique which more closely resembles the in vivo 3-D environment than plastic adherent monolayer cell culture (Paterson et al. 2020). Nevertheless, the synergistic effect of multiple inflammatory cytokines on tendon function in vitro is unknown.

Despite the evidence implicating inflammation in tendinopathy, there remains a lack of consensus surrounding inflammation-targeting treatments. Currently, the treatments showing the most promise include cellular therapies (Godwin et al. 2012; Gaspar et al. 2015), platelet-rich plasma (PRP) (Filardo et al. 2018), gene therapies (Tang et al. 2016) and pharmaceutical interventions which target specific inflammatory cytokines (Fredberg and Ostgaard 2009; Berkoff et al. 2016). Non-steroidal anti-inflammatory drugs (NSAIDs) which inhibit cyclooxygenase (COX) activity are often used initially following tendinopathy diagnosis to relieve pain (Wang et al. 2019). However, non-specific blockage of the inflammatory response using NSAIDs may inhibit tendon cell migration and proliferation and ultimately impair tendon healing (Marsolais et al. 2003). Therefore, for a tendinopathy treatment to be effective, it must be capable of diminishing the inflammatory response whilst still promoting tendon regeneration.

Bone marrow–derived mesenchymal stromal cells (BM-MSCs) are commonly used to treat equine tendon injuries. Clinical and experimental evidence shows BM-MSCs improve tendon healing and may be beneficial to reduce re-injury rates (Godwin et al. 2012; Smith et al. 2013). This may be due to BM-MSCs having regenerative and immunomodulatory properties (Paterson et al. 2014; Sullivan et al. 2014); however, their specific mechanism of action is largely unknown. The original concept was that BM-MSCs acted by direct differentiation into tenocytes due to their potential to differentiate into lineages of the mesenchyme such as osteoblasts, chondrocytes and adipocytes in vitro (Guest et al. 2008). However, the prolonged survival of BM-MSCs injected into injured equine tendon is poor (< 5% survival 10 days following implantation) (Guest et al. 2010; Becerra et al. 2013), suggesting BM-MSCs instead help tendon healing through trophic effects. BM-MSCs can modulate the inflammatory environment by influencing the activities of resident macrophages (Manning et al. 2015). Other studies suggest BM-MSCs can improve tendon healing through the secretion of soluble factors which protect endogenous cells from inflammation (Viganò et al. 2019). Consequently, it is apparent that further work is required to establish the exact role BM-MSCs have in improving tendon regeneration.

More targeted approaches to reduce inflammation in tendinopathy are under investigation. In vitro*,* blockage of IL-1β signalling using exogenous IL-1 receptor antagonist protein (IL-1RA) rescued the adverse consequences of IL-1β on 3-D collagen gel contraction by equine tenocytes (McClellan et al. 2019a). Nevertheless, IL-1RA is specific to IL-1β signalling and is unlikely to block the effects of the other inflammatory cytokines known to be upregulated in the injured tendon.

Inflammatory cytokines activate numerous signalling cascades including c-Jun N-terminal kinase (JNK), mitogen-activated protein kinases (MAPKs), nuclear factor kappa B (NF-κB) and signal transducer and activator of transcription (STATs) in musculoskeletal cell types (Tsai et al. 2014; McClellan et al. 2019a, b; Wang et al. 2019; Freedman et al. 2022). Furthermore, several animal models have implicated these inflammatory signalling pathways within tendon extracellular matrix interactions (Gupta et al. 2010; Schwartz et al. 2015; Abraham et al. 2017). Therefore, rather than targeting specific cytokines, it would be more beneficial to dissect the role inflammatory pathways such as NF-κB play in mediating tendon regeneration. This would allow the development of novel targeted therapeutic interventions which enhance tendon healing.

This in vitro study explored the effect a combination of IFN-γ, TNFα and IL-1β has upon equine tenocyte function and identified the key inflammatory signalling pathways which they activate. The effects of IL-1RA and BM-MSCs on attenuating adverse inflammatory responses to this combination of inflammatory cytokines were also determined.

## Materials and methods

### Study design

**Fig. 1:**
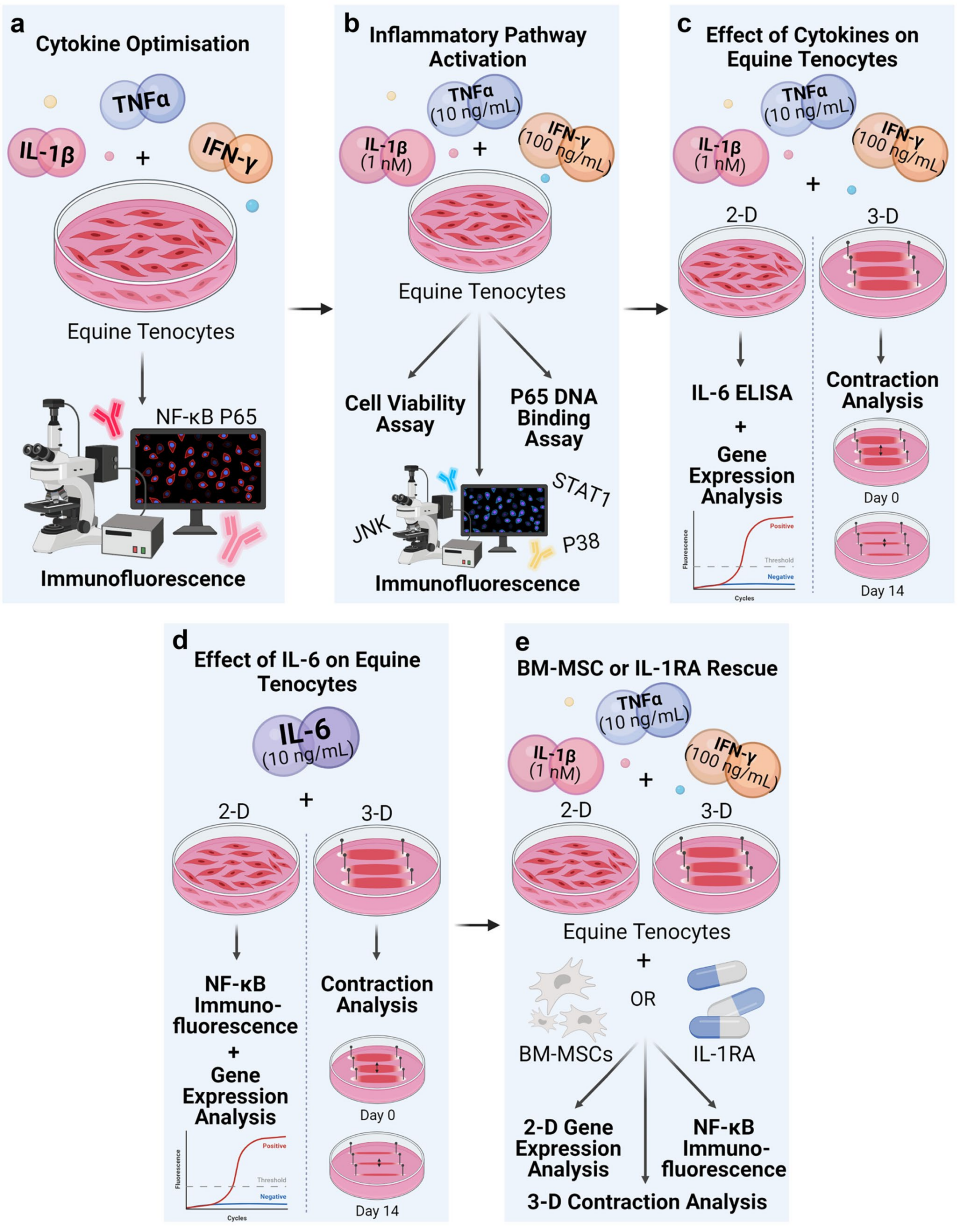
Experimental study design outlining cytokine optimisation (**a**), inflammatory pathway activation (**b**), effect of inflammatory cytokines on equine tenocytes (**c**), effect of IL-6 on equine tenocytes (**d**) and BM-MSC or IL-1RA rescue (**e**). Created with BioRender.com

An overview of the study design is provided in Fig. 1. Initial experiments measured NF-κB P65 nuclear translocation in equine tenocytes following exposure to different concentrations of IFN-γ, TNFα and IL-1β for various durations (Fig. 1a). The optimal dose and exposure time for maximal P65 translocation were then used to determine if there were any effects on cell viability and proliferation, confirm P65 DNA binding and determine if other signalling pathways (STAT1, JNK and P38 MAPK) were activated by measuring their nuclear translocation (Fig. 1b). Antibody specificity was confirmed using western blot. The effect of the cytokines on tenocyte 3-D collagen gel contraction, 2-D gene expression and IL-6 production was then determined (Fig. 1c). As IL-6 production was found to be increased following exposure to the inflammatory cytokines, the effect of IL-6 on P65 translocation, tenocyte 3-D collagen gel contraction and 2-D gene expression was determined (Fig. 1d). We then determined the effect of IL-1RA and BM-MSCs (co-culture and conditioned media) on attenuating adverse inflammatory responses by measuring their effects on cytokine mediated P65 translocation, tenocyte 3-D collagen gel contraction and 2D gene expression (Fig. 1e).

### Tendon cell isolation and culture

Tenocytes were harvested post-mortem from healthy SDFTs of a total of ten adult Thoroughbred horses euthanized for reasons unrelated to this study with approval from the Royal Veterinary College Clinical Research Ethical Review Board (URN 2020 2017–2). The horses used were aged between 2 and 13 years old: eight were males, and two were females. Tenocytes were isolated and cultured as described previously (Barsby and Guest 2013). Briefly, tendon tissue was dissected into pieces and incubated with 1 mg/mL type I collagenase from *Clostridium histolyticum* (Sigma-Aldrich, Dorset, UK) for 14–16 h at 37 °C. Dissociated cells were cultured in growth media consisting of Dulbecco’s modified Eagle’s medium (DMEM; high glucose [4500 mg/L] with sodium pyruvate [110 mg/L]) with 10% foetal bovine serum (FBS), 1% penicillin–streptomycin (P/S) and 2 mM L-glutamine (LQ) (all Gibco, Thermo Fisher, Hemel Hempstead, UK). Tenocytes were cultured at 37 °C in a humidified atmosphere of 5% CO_2_ and passaged before reaching maximum confluency every 3–4 days using 0.25% trypsin–EDTA (Sigma-Aldrich).

### Immunofluorescence

Three biological replicates of tenocytes (between P3 and P10) were cultured on gelatin-coated glass coverslips (Sigma-Aldrich; 70–80% confluent) prior to stimulation with recombinant human TNFα (25 pg/mL and 0.1, 1, 10 ng/mL) (Cheshire and Baldwin 1997; Lee et al. 2007; Tsai et al. 2014), recombinant human IL-1β (0.0425, 0.17, 1.7 and 17 ng/mL) (Gehwolf et al. 2019; McClellan et al. 2019a; Vinhas et al. 2020) and recombinant equine IFN-γ (1, 100, 200, 500 ng/mL) (Paterson et al. 2014; Yang et al. 2014; McClellan et al. 2019b) (all PeproTech, London, UK) alone or in combination for 20 min, 1 h, 2 h and 24 h. Unstimulated cells served as controls. Following stimulation, coverslips were fixed with 3% paraformaldehyde for 20 min. Fixed cells were permeabilised with 0.1% Triton-X-100 (Sigma-Aldrich) at room temperature for 1 h and then blocked with 2.5% normal horse serum (NHS) (Vector Laboratories, Peterborough, UK) for 20 min. Incubation with primary antibodies (see Table 1) was completed overnight at 4 °C in NHS, before detection with either goat anti-mouse IgG Alexa fluor 594 1:200 or goat anti-rabbit IgG Alexa Fluor 594 1:200 (both Thermo Fisher) in 2.5% NHS for 3 h at room temperature. Negative controls were performed using the secondary antibody only. Coverslips were mounted using Vectashield Hardset with DAPI (4′,6-diamidino-2-phenylindole, Vector Laboratories). Images were acquired using a Nikon Eclipse Ti2 series microscope (Nikon, Surrey, UK). Nuclear fluorescent intensity was quantified by measuring mean grey scale of the nucleus using ImageJ software.

**Table 1:** Primary antibodies used for Immunofluorescence

**Marker**	**Species**	**Clone**	**Dilution**	**Company**	**References**
NF-κB (P65)	Mouse	Monoclonal 572	1:100	Thermo Fisher (436,700)	Cross-reactivity shown by western blot (Fig. S4)
STAT1	Rabbit	Monoclonal EPR4407	1:200	Abcam, UK (ab109320)	Cross-reactivity shown by western blot (Fig. S4)
JNK 1,2,3	Rabbit	Monoclonal EPR16797-211	1:200	Abcam (ab179461)	Cross-reactivity shown by western blot (Fig. S4)
P38 MAPK	Rabbit	Polyclonal	1:100	Cell Signalling Technology, UK (CS9212)	Gardner et al. (2016)
TNF receptor 1	Rabbit	Polyclonal	1:100	Abcam (ab19139)	Fedorka et al. (2022)
TNF receptor 2	Rabbit	Monoclonal EPR1653	1:100	Abcam (ab109322)	
IFNGR1	Rabbit	Monoclonal EPR7866	1:100	Abcam (ab134070)	
IFNGR2	Rabbit	Polyclonal	1:100	Biorbyt, UK (ORB415521-BOR)	

### Western blot analysis

To confirm that the antibodies for NF-κB P65 (3 μg/mL), STAT1 (1:10,000) and JNK 1, 2 and 3 (1:1000) could recognise the equine protein, a western blot was performed. Whole cell protein extract (WCE) was isolated from equine skin fibroblasts (P5) by three rounds of freeze-thawing in a whole cell extraction buffer (20 mM Hepes pH 7.9, 450 mM NaCl, 0.4 mM EDTA, 25% glycerol, 1 mM PMSF), with supernatants collected by centrifugation. Twenty micrograms of reduced protein was then run on a TEO-Tricine Precast Gels, RunBlue^™^ 4–12% (Abcam) and transferred to a PDVF or nitrocellulose membrane. Immunoreactivity was detected using goat anti-mouse IgG H&L (HRP) (Abcam, ab6789, 1:10,000) or goat anti-rabbit IgG H&L (HRP) (Abcam, ab6721, 1:5000) and the ECL plus Western Blotting Substrate (Pierce^™^, Thermo Fisher).

### NF-κB activation assay

A DNA-binding ELISA was performed using three biological replicates of tenocytes between P6 and P10 that had been exposed to TNFα (10 ng/mL) (Cheshire and Baldwin 1997), IL-1β (17 ng/mL) (Tsuzaki et al. 2003; McClellan et al. 2019a) and/or IFN-γ (100 ng/mL) (Paterson et al. 2014) alone or in combination for 1 h. Unstimulated cells were used as controls. WCEs were isolated as described above. Accurate determination of protein concentration was determined using a Pierce BCA Protein Assay Kit (Thermo Fisher) and Tecan plate reader measuring absorbance at 562 nm (Infinite M Plex; Tecan, Switzerland). Protein samples were then subsequently analysed for NF-κB activation using the TransAM^™^ NF-κB P65 transcription factor assay kit (Active Motif, Belgium), according to the manufacturer’s instructions. Briefly, 20 μg of whole cell protein was added to each well of a 96-well plate containing a consensus NF-κB oligonucleotide and incubated for 1 h to allow the binding of P65 to the NF-κB consensus site. The presence of the resulting complex was detected using an NF-κB primary antibody, followed by a secondary antibody conjugated to horseradish peroxidase (HRP) which provided a sensitive colorimetric readout which could be quantified by spectrophotometry (Infinite M Plex; Tecan).

### Population doubling time and cell viability assay

The mean population doubling time (*DT*) was calculated from tenocytes in 2-D culture following 72 h stimulation with all combinations of TNFα (10 ng/mL), IL-1β (17 ng/mL) and/or IFN-γ (100 ng/mL). *DT* was calculated using the formula *DT* = *T* ln2/ln(*Xe*/*Xb*), where *T* = incubation time, *Xb* = starting cell number and *Xe* = ending cell number.

To measure differences in cell viability, tenocytes were seeded onto 96-well tissue culture plates at a concentration of 1.5 × 10^4^ cells/well in growth media and allowed to attach at 37 °C and 5% CO_2_ for 24 h. After 24 h, the tenocytes were stimulated with combinations of TNFα (10 ng/mL), IL-1β (17 ng/mL) and/or IFN-γ (100 ng/mL) for 72 h. After incubation, culture media was removed and 100 μL of diluted PrestoBlue^™^ reagent (1:10; Invitrogen, Thermo Fisher) was added to each well and the plates were incubated at 37 °C for 30 min. Fluorescence of each well was measured at an excitation wavelength of 560 nm and an emission of 590 nm on a Tecan plate reader (Infinite M Plex; Tecan, Switzerland). Both *DT* and cell viability assays were performed using three biological replicates of tenocytes (P4–P11).

### Three-dimensional cell culture of tenocytes

Three-dimensional (3-D) cellular culture in collagen gels was performed for 14 days as previously described (Barsby et al. 2014; Bavin et al. 2017; McClellan et al. 2019a, b; Paterson et al. 2020) using four biological replicates of tenocytes (P4–P10). Pairs of 0.2-mm-diameter minutien pins were embedded 15 mm apart into a silicone-coated six-well plate (Sylgard 184 Silicone elastomer; Corning) with each well containing three pairs of pins. Tenocytes (4 × 10^5^ cells/mL) were suspended in a chilled mixture of 2 parts growth medium and 8 parts PureCol (Bovine collagen type I; Advanced Biomatrix, Carlsbad, USA) with the pH adjusted to 7.2–7.6 with 1 M sodium hydroxide. Subsequently, 200 μL of collagen-cell suspension was pipetted between each pair of minutien pins. The plate was then parafilm sealed and kept at 37 °C for 60–90 min to allow the constructs to set. Once set, the 3-D constructs were cultured at 37 °C and 5% CO_2_, in growth media alone (no cytokine control) or containing combinations of TNFα (10 ng/mL), IL-1β (17 ng/mL), IFN-γ (100 ng/mL) and/or IL-1Ra (100 ng/mL) or IL-6 (10 ng/mL). Media was replenished every 3–4 days with new fresh cytokines as applicable.

The 3-D tendon constructs were photographed daily. Contraction analysis of these images was performed by measuring the average diameter of each gel using ImageJ software (National Institutes of Health, USA). Contraction data was displayed as a percentage of the day 0 value. To measure cell survival, constructs were harvested and digested in 1 mL of growth media with 1 mg/mL type I collagenase from *Clostridium histolyticum* (Sigma-Aldrich) for 20 min–1 h at 37 °C. Cell counts were performed using a haemocytometer, and results displayed as a percentage of the number of cells seeded on day 0. Collagen gel contraction and cell survival were performed using three to four biological replicates of tenocytes with each replicate containing 1 to 9 tendon-like constructs.

### RNA extraction, cDNA synthesis and quantitative PCR

Tenocytes from three biological replicates between P4 and P8 were used in these experiments. RNA was extracted using 1 mL Tri-reagent (Sigma-Aldrich) per 10 cm plate, or 3 wells of a 6-well plate, of 2-D cultured cells. RNA was next purified using the Qiagen RNeasy kit (Qiagen, Manchester, UK) and contaminating genomic DNA removed using a DNA-free^™^ DNA removal kit (Invitrogen, Thermo Fisher) according to the manufacturer’s instructions. RNA concentrations were calculated using a DeNovix Spectrophotometer (DeNovix, Wilmington, USA), ensuring the 260:280 ratio was between 1.8 and 2.2. cDNA was synthesized from 1 μg of RNA using the sensiFAST™ cDNA synthesis kit (Bioline, London, UK). Equine-specific primers were designed using primer3 (http://primer3.ut.ee) and mfold (http://unafold.rna.albany.edu/?q=mfold) to obtain amplicons with a melting temperature (Tm) of 58–62 °C, lacking a secondary structure at Tm 60 °C, and with an amplicon size of 50–150 bp. Primer sequences can be found in Table 2. Forty nanograms of cDNA was used for each qPCR reaction with a SYBR Green containing supermix (Bioline) on a Bio-Rad C1000 Touch Thermal Cycler (Bio-Rad, Hertfordshire, UK) and performed in duplicate. PCR cycle parameters were as follows: 95 °C (10 min), followed by 45 cycles of 95 °C (15 s), 60 °C (15 s) and 72 °C (15 s). Following this, a melt curve was produced with readings taken every 1 °C from 65 to 95 °C. Analysis of three candidate reference genes (*18 s rRNA*, glyceraldehyde 3-phosphate dehydrogenase (*GAPDH*) and *β-actin*) was performed using the comprehensive gene ranking program RefFinder https://www.heartcure.com.au/reffinder/ (Xie et al. 2012) (data not shown). Consequently, relative gene expression levels were normalised with the housekeeping gene *18 s rRNA* using the 2^−ΔΔCt^ method (Livak and Schmittgen 2001). qPCR data was presented as fold change in gene expression of the inflammatory cytokine treated cells, compared with untreated controls.

**Table 2:** Primer sequences used for gene expression analysis

**Gene**	**Protein name**	**Gene ID**	**Forward primer**	**Reverse primer**
*18S rRNA*	18 s ribosomal RNA	100861557	CCCAGTGAGAATGCCCTCTA	TGGCTGAGCAAGGTGTTATG
*GAPDH*	Glyceraldehyde 3-phosphate dehydrogenase	100033897	CGACCACTTTGTCAAGCTCA	GTCCACCACCCTATTGCTGT
*β-Actin*	Actin, cytoplasmic 1	100033878	CCAGCACGATGAAGATCAAG	GTGGACAATGAGGCCAGAAT
*SCX*	Basic helix-loop-helix transcription factor scleraxis	100125857	CCCAAACAGATCTGCACCTT	ATCCGCCTCTAACTCCGAAT
*TNC*	Tenascin	100049835	AACCCGTCCAAAGAGACCTT	GCGTGGGATGGAAGTATCAT
*COL1A1*	Collagen alpha-1(I) chain	100033877	TGCGAAGACACCAAGAACTG	GACTCCTGTGGTTTGGTCGT
*COL3A1*	Collagen type III alpha 1 chain	100034123	CTGGTGCTAATGGTGCTCCT	TCTCCTTTGGCACCATTCTT
*COMP*	Cartilage oligomeric matrix protein	100033911	AGAACATCATCTGGGCCAAC	CGCTGGATCTCGTAGTCCTC
*THBS4*	Thrombospondin-4	100073261	GGGAAATGGGGTTACCTGTT	CGGGTAGCAGGGATGATATT
*SOX9*	Transcription factor SOX-9	100033908	GCTCTGGAGACTTCTGAACGA	GTAATCCGGGTGGTCCTTCT
*MMP1*	Matrix metallopeptidase 1 (interstitial collagenase)	100033896	CTTTGATGGACCTGGAGGAA	GAATGGCCAAATTCATGAGC
*MMP2*	Matrix metallopeptidase 2 (72 kDa type IV collagenase)	100033948	CAGGAGGAGAAGGCTGTGTT	AGGGTGCTGGCTGAGTAGAC
*MMP3*	Matrix metallopeptidase 3 (stromelysin-1)	100034195	TGGACCTGGAAAAGTTTTGG	GACCAAGTTCATGAGCAGCA
*MMP8*	Matrix metallopeptidase 8 (neutrophil collagenase)	100069005	TTTGATGGACCCAATGGAAT	TTCATGGGCAGCAACAATAA
*MMP9*	Matrix metallopeptidase 9	100056599	GAGATCGGGAATCATCTCCA	CCAAGAGTCGCCAGTACCTC
*MMP13*	Matrix metallopeptidase 13 (collagenase 3)	100009711	GCCACTTTGTGCTTCCTGAT	CGCATTTGTCTGGTGTTTTG

### IL-6 ELISA

The IL-6 concentrations in conditioned media obtained from three biological replicates of tenocytes (between P4 and P8) stimulated with or without inflammatory cytokines for 72 h were measured by an equine IL-6 ELISA kit with an additional ancillary kit (both R&D systems) according to the manufacturer’s instructions. Each biological replicate was measured in duplicate on a Tecan plate reader (Infinite M Plex; Tecan). Colorimetric detection was performed at 450 nm with a background correction read at 540 nm. Optical density values were converted to picograms per microliter concentrations utilising a seven-point standard curve and data reduction with four-parameter logistic regression.

### IL-6 treatment of equine tenocytes

As IL-6 production was found to be increased following exposure to the inflammatory cytokines, the effect of IL-6 on P65 translocation, tenocyte 3-D collagen gel contraction and 2-D gene expression was determined. Three biological replicates of tenocytes between P2 and P9 were used in these experiments and exposed to 10 ng/mL (John et al. 2010; Martincuks et al. 2017) of recombinant human IL-6 (PeproTech). Unstimulated cells served as controls.

### IL-1RA treatment of equine tenocytes

We determined the effect of IL-1RA on attenuating adverse inflammatory responses by measuring their effects on cytokine mediated P65 translocation, tenocyte 3-D collagen gel contraction and 2-D gene expression. Three (four for 3-D culture experiments) biological replicates of tenocytes between P4 and P10 were used in these experiments and exposed to either IL-1β (17 ng/mL) alone or a combination of IL-1β (17 ng/mL), TNFα (10 ng/mL) and IFN-γ (100 ng/mL) in the presence or absence of 100 ng/mL recombinant human IL-1RA (Sigma-Aldrich) (McClellan et al. 2019a). Unstimulated cells and cells treated with IL-1RA alone were used as controls. P65 translocation was measured after 1 h of cytokine exposure, 2-D gene expression after 72 h of cytokine exposure and 3-D collagen gel contraction over 14 days of cytokine exposure (all as described in the sections above).

### BM-MSC culture

BM-MSCs were derived from bone marrow aspirates retrieved from a total of four Welsh mountain ponies immediately following euthanasia for reasons unrelated to this study with the approval of the Animal Health Trust Ethical Review Committee (AHT_02_2012). The ponies were aged between 2 and 5 years old. Three were male, and one was female. All BM-MSCs used were formally processed and cryopreserved as described previously (Guest et al. 2008; Paterson et al. 2014). Cells were used with approval of the Royal Veterinary College Clinical Research Ethical Review Board (URN 2021 2035–2). BM-MSCs were cultured in growth media (as detailed above) at 37 °C and 5% CO_2_. Medium was replenished every 2–3 days, and the cells passaged using 0.25% trypsin–EDTA (Sigma-Aldrich) every 3–7 days prior to reaching maximum confluency. BM-MSC-conditioned media was obtained by culturing the BM-MSCs on 10 cm^2^ plates until 70–80% confluent. The culture media was then removed and replenished with fresh growth media which was collected after 48 h of incubation. This “BM-MSC-conditioned media” was passed through a 0.22-μm filter (Sigma-Aldrich) to remove cell debris and was used immediately.

### Flow cytometry

Flow cytometry was performed using two biological replicates of BM-MSCs between P5 and P7 (Guest et al. 2022). BM-MSCs were fixed with 3% paraformaldehyde for 20 min and then washed three times with phosphate-buffered saline (PBS). Blocking was carried out using 2.5% NHS for 20 min and 1 × 10^6^ cells were incubated with primary antibodies (see Table 3) or corresponding concentrations of isotype control (mouse IgG, Vector Laboratories) for 45 min at 4 °C. Following washing with PBS, the BM-MSCs were incubated with goat-anti mouse FITC (1:200 dilution, ab7064, Abcam) for 45 min at 4 °C and then washed three times with PBS. A maximum of 10,000 events were acquired on a flow cytometer (BD LSRFortessa x-20 4 laser system, BD Biosciences). The data was subsequently analysed using FlowJo (version 10.8.1). Events were gated using forward versus side scatter of unlabelled cells to exclude dead cells and debris (R1). These events were then gated using forward scatter height versus scatter area to remove doublets (R2). Two percent of the remaining events in the isotype control and everything to the right were included in a new gate (R3) by FITC area versus forward scatter height. The overlayed antibody events gated within the corresponding isotype controls R3 gate were considered positive.

**Table 3:** Primary antibodies used for flow cytometry

**Marker**	**Species**	**Clone**	**Concentration**	**Company**	**References**
CD90	Mouse	Monoclonal 5E10	625 ng/mL	BD Biosciences, Berkshire, UK (550402)	Guest et al. (2008)
CD29	Mouse	Monoclonal 4B4LDC9LDH8	10 μg/mL	Beckman Coulter, High Wycombe, UK (6603113)	Guest et al. (2008)
CD45	Mouse	Monoclonal F10-89–4	5 μg/mL	Bio-Rad (MCA87T)	Spaas et al. (2013); Gomiero et al. (2016)
CD14	Mouse	Monoclonal M5E2	5 μg/mL	BD Biosciences (557152)	Guest et al. (2008)

### BM-MSC and tenocyte co-culture in 2-D

Two-dimensional (2-D) BM-MSC/tenocyte co-culture experiments were performed using three biological replicates of BM-MSCs (P4–P5) and four biological replicates of tenocytes (P3–P10). Six-well transwell culture plates with a 0.4-μm membrane pore size (Corning, Flintshire, UK) were used to physically separate the BM-MSCs and tenocytes. Tendon cells (2 × 10^5^ cells/well) were seeded into each well (outer chamber) of a transwell culture plate and allowed to attach at 37 °C and 5% CO_2_ for 24 h. For immunofluorescence, each well was lined with gelatin-coated (Sigma-Aldrich) glass coverslips prior to seeding tenocytes. After 24 h, BM-MSCs which had been mitotically inactivated for 2 h with 10 μg/mL mitomycin C (MMC) from *Streptomyces caespitosus* (Sigma-Aldrich) were seeded into the inner chamber of each well (1 × 10^5^ cells/well). Growth media alone (no cytokine control) or containing TNFα (10 ng/mL), IL-1β (17 ng/mL) and IFN-γ (100 ng/mL) was then added to the inner and outer chamber of each well. For immunofluorescence, tenocytes seeded on glass coverslips were fixed at room temperature using 3% paraformaldehyde (Sigma-Aldrich) after 1 h of adding the BM-MSCs. This time point was optimised in the earlier experiments for immunofluorescent analysis of P65 nuclear translocation (as described earlier). For qPCR, RNA was harvested from tenocytes using Tri-reagent (Sigma-Aldrich) after 72 h of co-culture (as optimised previously (McClellan et al. 2019a) and used in the earlier experiments above). In addition to the no cytokine controls but the presence of BM-MSCs, tenocytes cultured in the absence of BM-MSCs were used as controls (with or without the presence of inflammatory cytokines).

### BM-MSC-conditioned media and tenocyte co-culture in 2-D

BM-MSC-conditioned media/tenocyte co-culture experiments were performed using three biological replicates of BM-MSCs (P4–P5) and four biological replicates of tenocytes (P3–P10). Tenocytes (2 × 10^5^ cells/well) were seeded into 6-well plates and allowed to attach at 37 °C and 5% CO_2_ for 24 h. For immunofluorescence, 24-well plates were lined with gelatin-coated (Sigma-Aldrich) glass coverslips prior to seeding tenocytes. After 24 h, previously prepared 48-h BM-MSC-conditioned media alone (no cytokine control) or containing TNFα (10 ng/mL), IL-1β (17 ng/mL) and IFN-γ (100 ng/mL) was then added to each well of tenocytes. For immunofluorescence, tenocytes seeded on glass coverslips were fixed at room temperature using 3% paraformaldehyde (Sigma-Aldrich) after 1 h of adding the BM-MSC-conditioned media. This time point was optimised in the earlier experiments (see above) for immunofluorescent analysis of P65 nuclear translocation (as described previously). For qPCR, RNA was harvested from tenocytes using Tri-reagent (Sigma-Aldrich) after 72 h of culture. In addition to the no cytokine controls but the presence of conditioned media, tenocytes cultured in the absence of BM-MSC-conditioned media were used as controls (with or without the inflammatory cytokines).

### BM-MSC/BM-MSC-conditioned media and tenocyte co-culture in 3-D

3-D co-culture experiments were performed using three biological replicates of BM-MSCs (P4–P5) and four biological replicates of tenocytes (P4–P6). Pairs of minutien pins were inserted into silicone inserts (2 cm × 2 cm square) produced by punching a silicone-coated 10-cm plate with a metal cutter. Individual inserts were placed centrally into each well of a 6-well plate and the prepared collagen-tenocyte suspension seeded as previously described with each insert holding two sets of pins. Once the constructs had set, 4 mL of growth media (with or without cytokines as described above) was added to the wells with or without 2.5 × 10^5^ MMC mitotically inactivated BM-MSCs and maintained at 37 °C and 5% CO_2_ for 14 days. BM-MSCs attached to the tissue culture surface around the outside of the silicone inserts. BM-MSC morphology was monitored daily, with media replaced every 3–4 days. On day 14, BM-MSCs were fixed with 3% paraformaldehyde (Sigma-Aldrich) for 20 min prior to staining with 1% Crystal Violet solution (Sigma-Aldrich) for 20 min to observe cell distribution. Alternatively, BM-MSC-conditioned media was used, collected as described earlier. In both cases, tenocyte cell viability and the degree of tenocyte collagen gel contraction was measured as described in the previous section “Three-dimensional cell culture of tenocytes”.

### Statistical analysis

Statistical analysis was performed using Excel (Microsoft^™^) and SPSS (version 28.0.0.0.; IBM, UK). All data sets were tested to ensure Gaussian distribution using the Shapiro-Wilks normality test and equal variance using Levene’s test. For comparisons of two groups, independent Student’s *t*-tests were used. Data with more than two groups was analysed using a one-way ANOVA with post hoc Tukey. Where data was not normally distributed, a non-parametric Kruskal Wallis test was performed followed by Dunn’s pairwise comparisons. In all cases, a *p* value of < 0.05 was considered statistically significant.

## Results

### IL-1β and TNFα, but not IFN-γ, activate NF-κB P65 in tenocytes

**Fig. 2:**
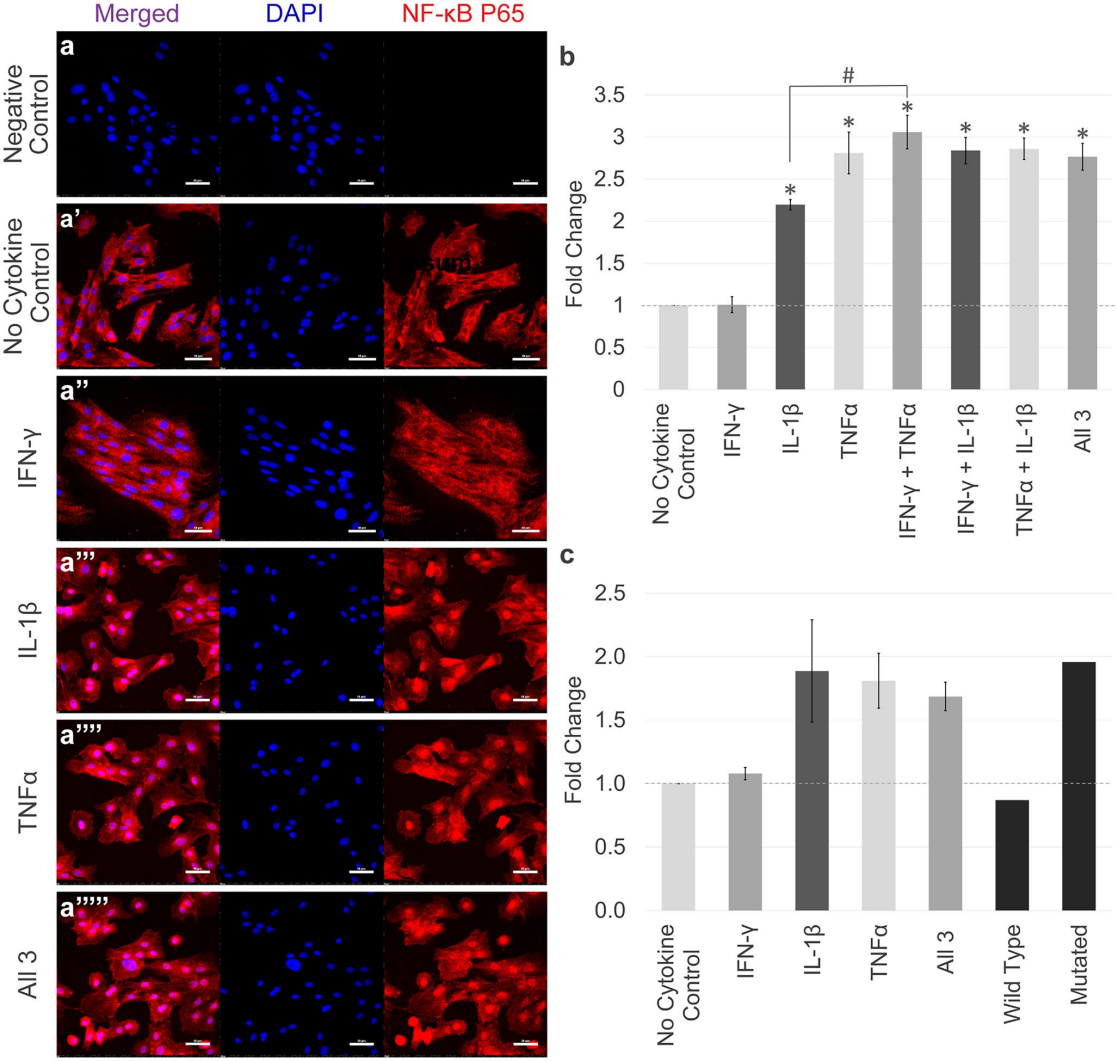
The localisation and DNA binding of NF-κB P65 in response to inflammatory cytokine stimulation. (**a**–**a**’’’’’) Immunofluorescence staining of NF-κB P65 in tenocytes following 1 h of IL-1β, TNFα and/or IFN-γ stimulation. DAPI staining of the nucleus is shown in blue. Images are representative of three biological replicates. Scale bar = 50 µm. (**b**) Quantification of the relative nuclear fluorescent intensity of NF-κB P65 immunofluorescence following 1 h of cytokine stimulation. Data shown as fold change compared to the no cytokine control. Error bars represent the S.E.M of three measurements from each of three biological replicates. Asterisk indicates the fold change in nuclear fluorescent intensity is significantly different to the no cytokine control and IFN-γ (*p* < 0.05). Number sign indicates the fold change in nuclear fluorescent intensity is significantly different between conditions (*p* < 0.05). (**c**) NF-κB P65 DNA binding ELISA following 1 h of cytokine stimulation. Results are also shown from combining one biological replicate of tenocytes stimulated with all three cytokines with a wild-type consensus oligonucleotide (competitor of DNA binding) and a mutated consensus oligonucleotide (no effect on DNA binding). Error bars represent the S.E.M of three biological replicates. Cells in these experiments were between P3 and P10

Initially, dose and time response experiments were carried out to establish optimal inflammatory cytokine conditions (Fig. S1). At these optimal concentrations, there was no significant effect of the cytokines on tenocyte proliferation or viability (Fig. S2). Furthermore, unstimulated tenocytes expressed the receptors for IL-1β (McClellan et al. 2019a), IFN-γ and TNFα at the protein level (Fig. S3). Immunofluorescence demonstrated that IL-1β and TNFα, but not IFN-γ, induced significant P65 nuclear translocation compared to the no cytokine control within 1 h of stimulation (Fig. 2a–a’’’’’ and b). A further significant difference in the amount of nuclear P65 was evident between IL-1β alone and TNFα in combination with IFN-γ, but none of the other combinations (Fig. 2b). To confirm that P65 nuclear translocation resulted in P65 DNA binding activity, a DNA-binding ELISA was performed. IFN-γ failed to induce P65 DNA binding, whereas treatment with TNFα and IL-1β individually, and all 3 cytokines in combination, led to a 1.6- to 1.8-fold induction of P65 DNA binding compared to the no cytokine control (Fig. 2c). Although these increases were not statistically significant, a binding-site competitor oligonucleotide was able to reverse the trend induced by all three cytokines suggesting binding was occurring.

### Nuclear translocation of STAT1, JNK and P38 MAPK in response to IL-1β, TNFα and IFN-γ stimulation in tenocytes

**Fig. 3:**
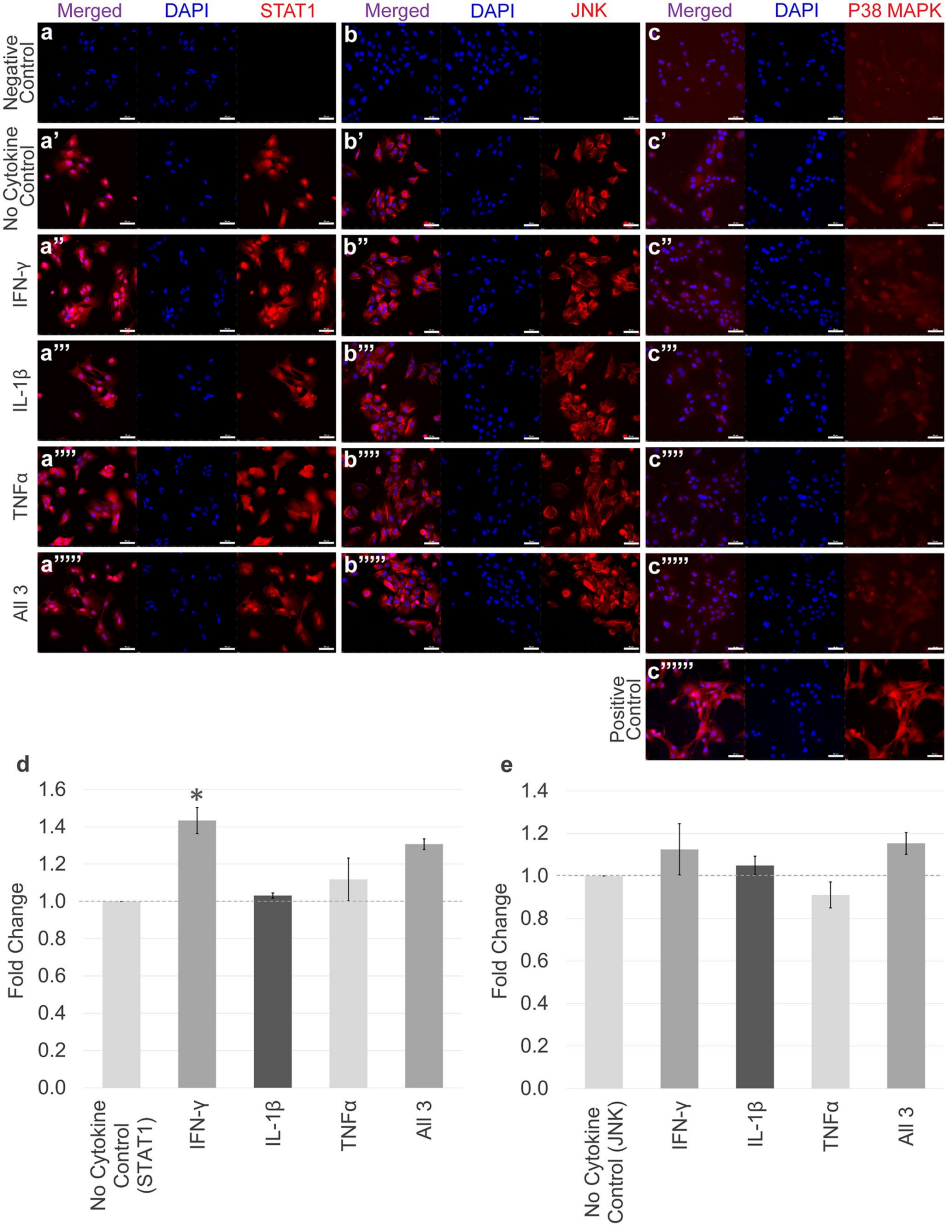
The localisation and activation of other inflammatory pathways in response to inflammatory cytokine stimulation. Immunofluorescence staining of STAT1 (**a**–**a**’’’’’), JNK (**b**–**b**’’’’’) and P38 MAPK (**c**–**c**’’’’’’) in tenocytes following IL-1β, TNFα and/or IFN-γ stimulation. c’’’’’’ shows staining of equine induced pluripotent stem cells and acts as a positive control. DAPI staining of the nucleus is shown in blue. All images are representative of three biological replicates. Scale bar = 50 µm. Quantification of the relative nuclear fluorescent intensity of STAT1 (**d**) and JNK (**e**) following cytokine stimulation is also shown. Data shown as fold change compared to the no cytokine control. Error bars represent the S.E.M of three measurements from each of three biological replicates. Asterisk indicates the fold change in nuclear fluorescent intensity is significantly different to the no cytokine control (*p* < 0.05). Cells in these experiments were between P4 and P8

Immunofluorescent staining showed basal nuclear STAT1 localisation in tenocytes under no cytokine control conditions (Fig. 3a–a’’’’’) which was significantly increased by IFN-γ, but not by TNFα and IL-1β or a combination of all three cytokines (Fig. 3d). In contrast, immunofluorescent staining for JNK did not demonstrate activation by any cytokine combination (Fig. 3b–b’’’’’ and e). The P38 MAPK signalling pathway was also investigated; however, minimal P38 MAPK was visualised in equine tenocytes (Fig. 3c–c’’’’’’). We confirmed the P38 MAPK antibodies cross-reactivity, previously shown to cross-react with equine cells (Gardner et al. 2016), by demonstrating positive staining in spontaneously differentiated equine induced pluripotent stem cells. Additionally, P38 MAPK has formally been shown to be expressed at the mRNA level in equine tenocytes (Paterson et al. 2020). Western blot analysis confirmed that the antibodies used to detect NF-κB P65, JNK and STAT1 recognise the specific equine proteins (Fig. S4).

### IL-1β, TNFα and IFN-γ work synergistically to inhibit 3-D collagen gel contraction by tenocytes

**Fig. 4:**
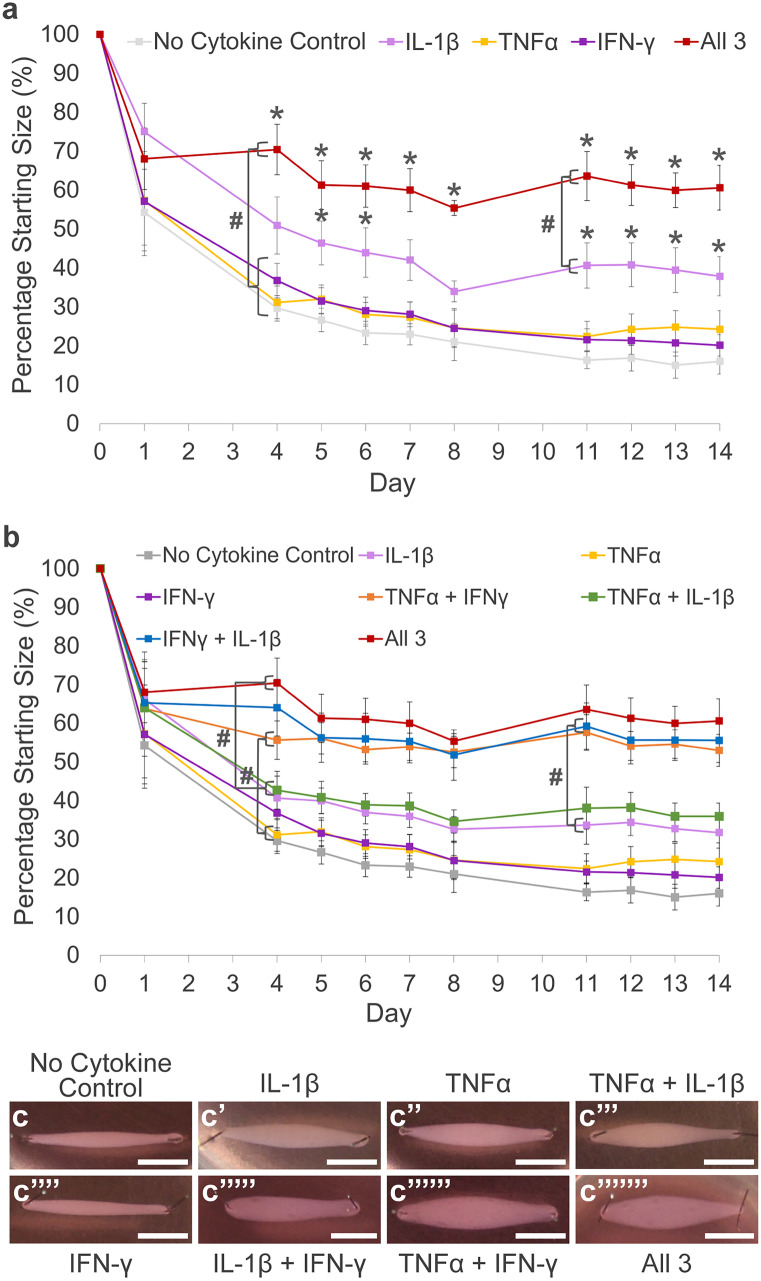
The effect of inflammatory stimulation on 3-D collagen gel contraction by tenocytes. (**a**) Inflammatory cytokines used in combination reduce collagen gel contraction by tenocytes to a greater extent than when used individually. Asterisk indicates the degree of collagen gel contraction is significantly different to the no cytokine control (*p* < 0.05). Number sign indicates the degree of collagen gel contraction is significantly different to all 3 cytokines in combination (*p* < 0.05). (**b**) IFN-γ potentiates the effect of TNFα and IL-1β on 3-D collagen gel contraction by tenocytes. Number sign indicates the degree of collagen gel contraction is significantly different between indicated conditions at *p* < 0.05. All error bars represent the S.E.M of four independent biological replicates of tenocytes. Representative images of 3-D collagen gel contraction at day 14 are shown in c–c’’’’’’’. Scale bar = 5 mm. Cells in these experiments were between P4 and P10

The degree of 3-D collagen gel contraction by tenocytes was significantly impaired by IL-1β, while TNFα and IFN-γ alone showed no significant effect (Fig. 4a). However, when applied in combination, all three cytokines caused the greatest impairment in 3-D collagen gel contraction, with significant differences to the no cytokine control, TNFα alone and IFN-γ alone from day 4 onwards, and to IL-1β from day 11 onwards, suggesting a synergistic effect. Whilst IFN-γ alone had little effect on 3-D collagen gel contraction, we show that it can potentiate the effects of the other cytokines (Fig. 4b and c–c’’’’’’’). In combination, IFN-γ with TNFα demonstrated a significantly greater inhibitory effect on 3-D collagen gel contraction when compared to TNFα alone from day 4 onwards. IFN-γ with IL-1β showed a significantly greater inhibitory effect on 3-D collagen gel contraction when compared to IL-1β alone but from day 11 onwards. The inhibitory effect on collagen gel contraction was also significantly greater when IFN-γ was added to TNFα and IL-1β from day 4 onwards. Differences in 3-D collagen gel contraction were not due to cytokine effects on tenocyte cell survival which show no significant differences at day 14, although there is a trend for the combination of all three cytokines to reduce survival compared to the no cytokine control (Fig. S5a). Furthermore, the contraction ability of the 3-D collagen gels is not due to the collagen itself as cell-free constructs fail to contract over 14 days (Fig. S5b).

### Inflammatory cytokines alter tendon-associated gene expression in tenocytes

**Fig. 5:**
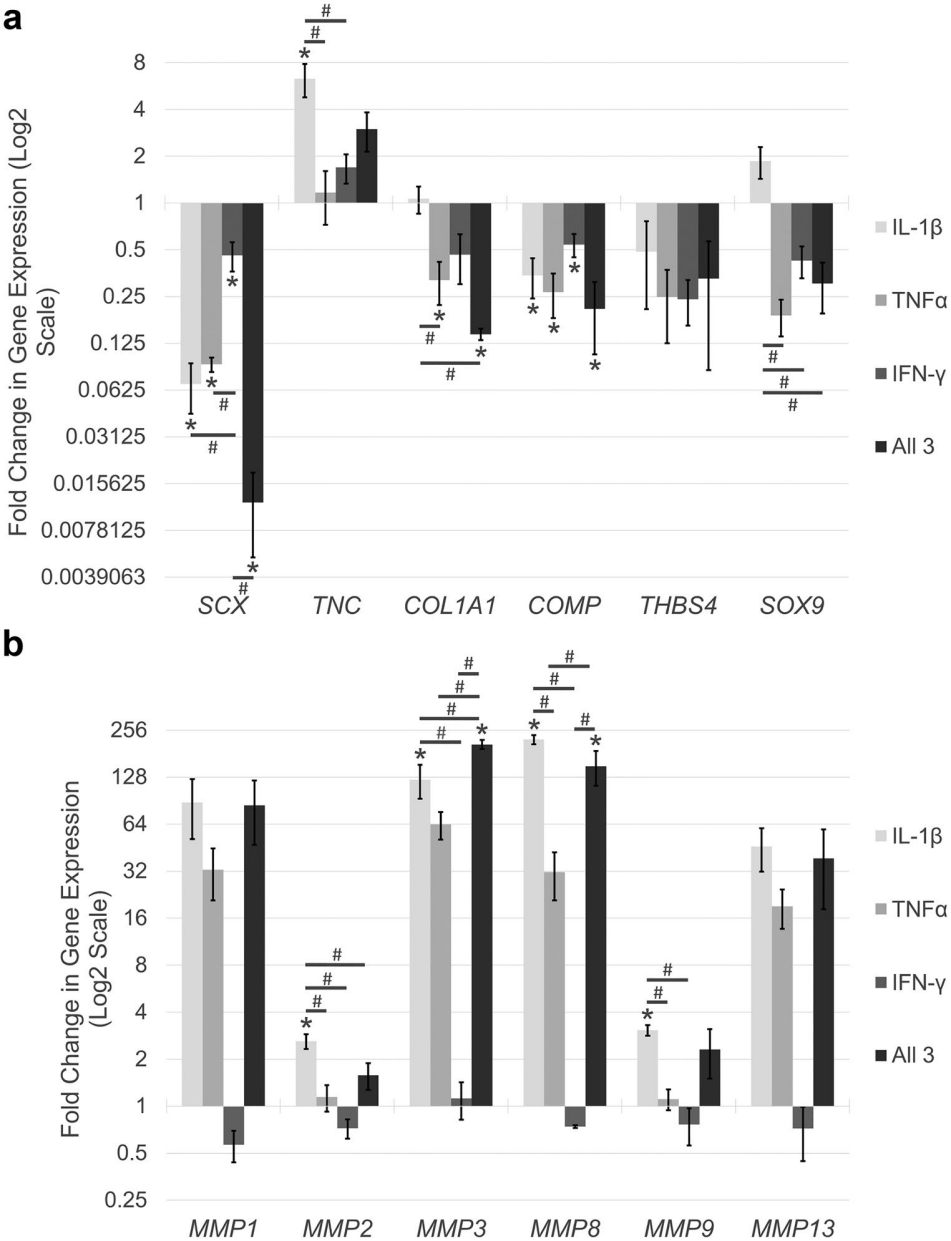
Inflammatory cytokines alter gene expression in tenocytes. Fold change in tendon-associated (**a**) and *MMP* (**b**) gene expression in tenocytes following stimulation with IFN-γ, TNFα and/or IL-1β compared to the no cytokine control. Asterisk indicates significant difference relative to no cytokine control *p* < 0.05. Number sign indicates a significant difference between indicated cytokine conditions (*p* < 0.05). Error bars represent the S.E.M of three biological replicates. Cells in these experiments were between P4 and P8

qPCR analysis was performed to examine changes in tendon-associated gene expression in tenocytes following 72 h simulation with IFN-γ, TNFα and/or IL-1β (Fig. 5a). Treatment of tenocytes with IL-1β, TNFα or IFN-γ alone or all three cytokines in combination resulted in significant downregulation of *SCX*, *COL1A1* and *COMP* expressions*.* In contrast, *TNC* was upregulated, although this was only significant for IL-1β. The only significantly potentiating effect of adding IFN-γ was for *COL1A1,* which was significantly downregulated when IFN-γ was combined with IL-1β, compared to IL-1β alone (data not shown). There was a tendency for all 3 cytokines in combination to have a greater effect in altering some tendon-associated gene expression than when the cytokines were used individually; however, this was not significant for any gene examined.

### IL-1β and TNFα, but not IFN-γ, increase MMP gene expression in tenocytes

Stimulation with IFN-γ alone had minimal effects in altering *MMP* gene expression in tenocytes, whereas stimulation of tenocytes with IL-1β alone, TNFα alone and all three cytokines in combination resulted in substantial increases in *MMP* expression (Fig. 5b). In general, TNFα produced smaller changes in *MMP* expression, exhibiting little impact on *MMP2* and *MMP9* expression and upregulating *MMP1, MMP3* and *MMP13* to a lesser degree than IL-1β alone and all three cytokines in combination. Despite the *MMPs* being consistently upregulated in all replicates, due to the variation in the fold increase between biological replicates, not all changes were statistically significant (see Fig. 5 for significantly different levels).

### IL-6 production is induced by exposure to IL-1β, but IL-6 alone does not activate NF-κB signalling and has no effect on 3-D collagen gel contraction or gene expression by tenocytes

**Fig. 6:**
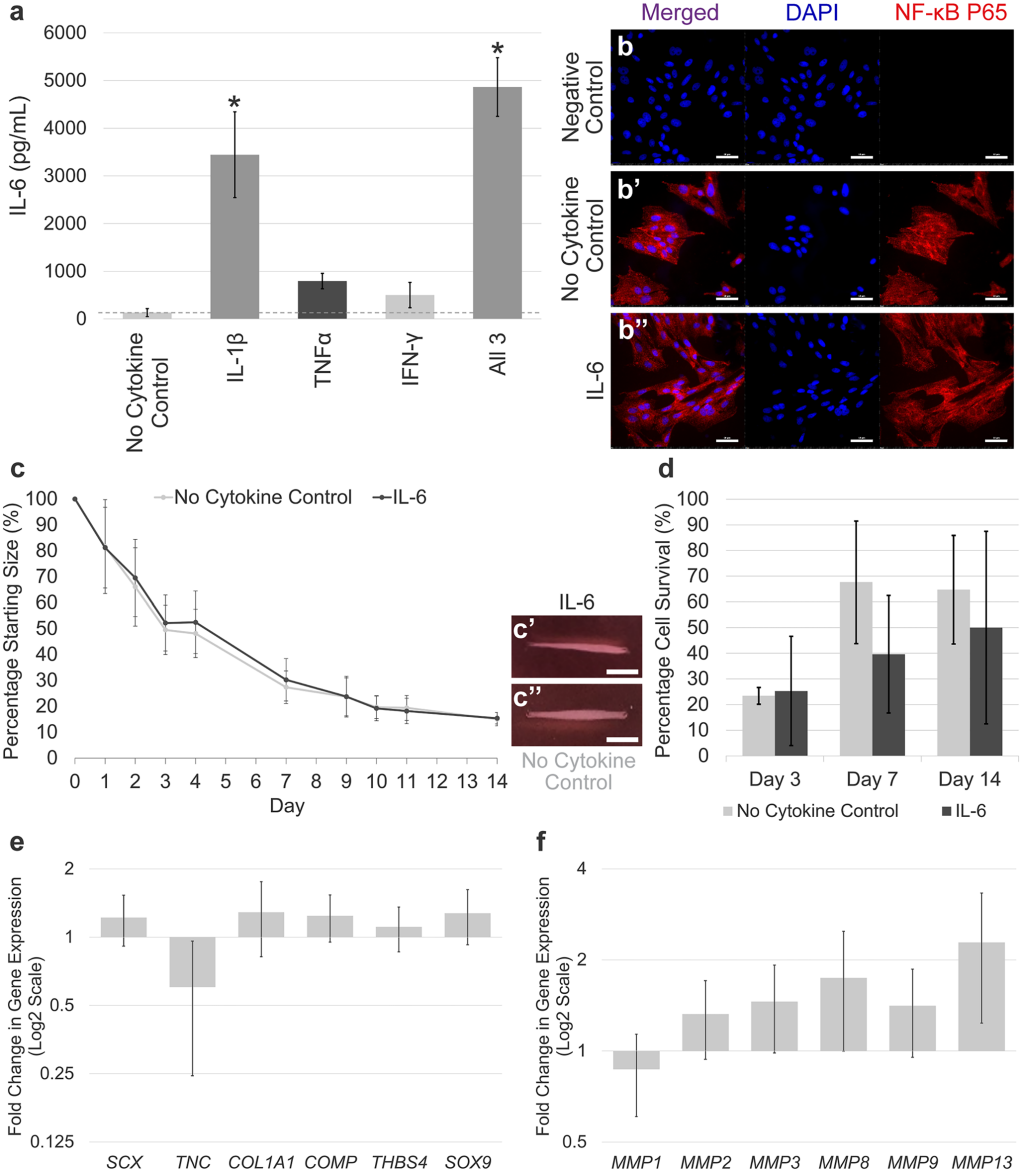
IL-6 fails to activate NF-κB signalling and exhibits no effect on 3-D collagen gel contraction or gene expression by tenocytes. (**a**) Secretion of IL-6 by tenocytes is significantly increased by IL-1β and all three cytokines in combination, but not by TNFα or IFN-γ alone. (**b**–**b**’’) Immunofluorescence staining of NF-κB P65 in tenocytes following 1 h of IL-6 stimulation. DAPI staining of the nucleus is shown in blue. Images are representative of three biological replicates. Scale bar = 50 µm. (**c**) IL-6 has no effect on 3-D collagen gel contraction by tenocytes. Representative images of gel contraction at day 14 are shown (**c**’–**c**’’). Scale bar = 5 mm. (**d**) No significant differences in cell survival were found between IL-6 and no cytokine control gels. Fold change in tendon-associated (**e**) and *MMP* (**f**) gene expression in tenocytes following stimulation with IL-6 compared to the no cytokine control. Each experiment was performed using three biological replicates. Asterisk indicates significant difference compared to the no cytokine control (*p* < 0.05). Error bars represent the S.E.M of three biological replicates. Cells in these experiments were between P2 and P9

Secretion of IL-6 by tenocytes was significantly increased by IL-1β alone and all three cytokines in combination, whereas stimulation with TNFα alone and IFN-γ alone caused minimal increases in IL-6 secretion (Fig. 6a). The impact of IL-6 on responses of tenocytes in vitro was also investigated. Immunofluorescence demonstrated 1 h of IL-6 stimulation did not simulate NF-κB P65 nuclear translocation in tenocytes (Fig. 6b–b’’). Further stimulation timepoints were examined but no NF-κB P65 nuclear translocation was seen (data not shown). In 3-D culture, IL-6 exhibited no significant inhibition in the final degree of 3-D collagen gel contraction (Fig. 6c–c’’) or tenocyte survival (Fig. 6d) compared to the no cytokine control. Finally, IL-6 had minimal effects in altering tendon-associated and *MMP* gene expression (Fig. 6e and f).

### IL-1RA fails to protect tenocytes when IL-1β, TNFα and IFN-γ are used in combination

**Fig. 7:**
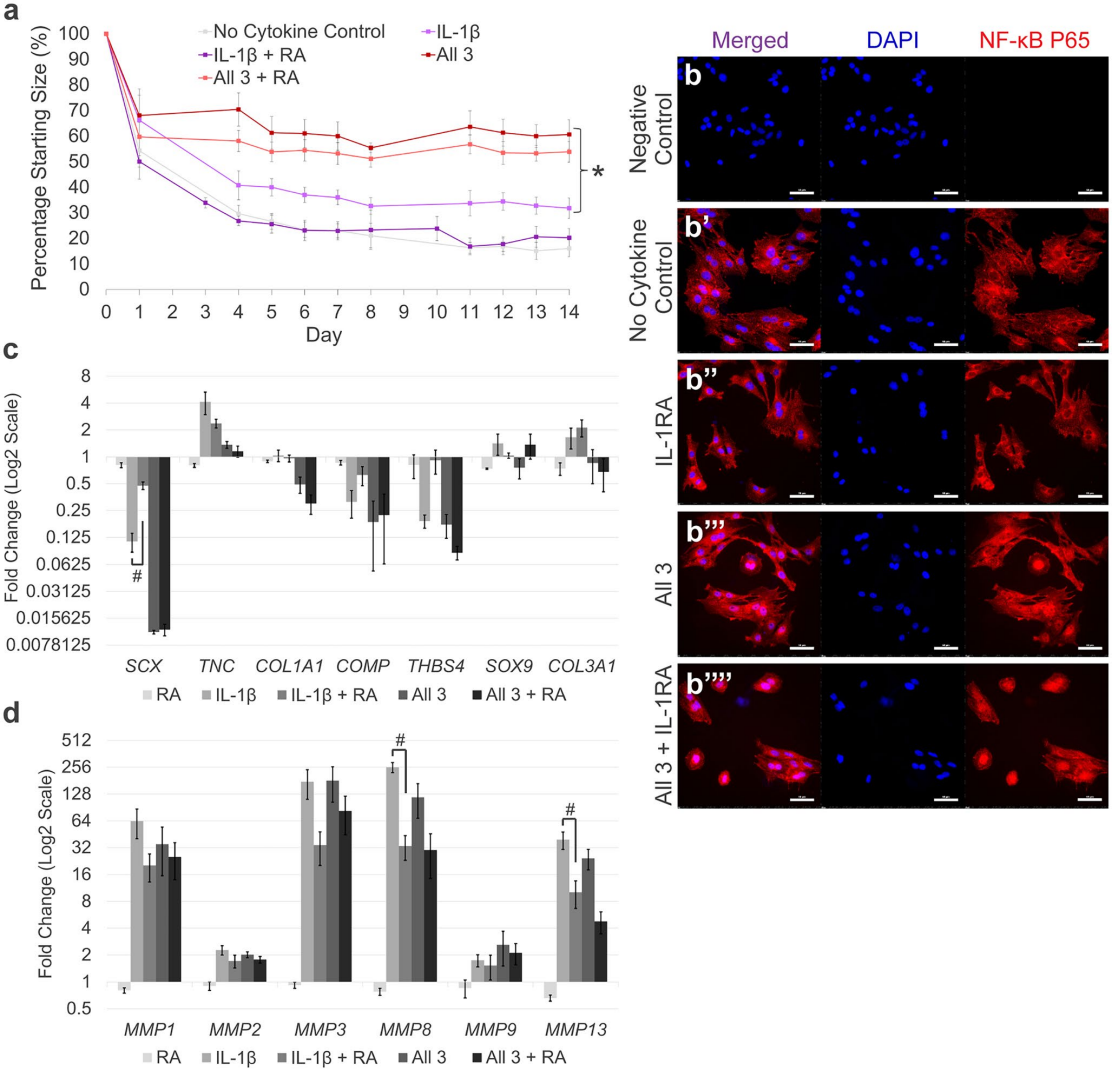
IL-1RA fails to protect tenocytes when stimulated with IL-1β, TNFα and IFN-γ in combination. (**a**) IL-1RA (RA) is able to block the effects of IL-1β alone, but not the effects of a combination of all three cytokines on 3-D collagen gel contraction by tenocytes. Asterisk indicates the final degree of collagen gel contraction is significantly different to the no cytokine control (*p* < 0.05). Error bars represent the S.E.M of four biological replicates of tenocytes. (**b**–**b**’’’’) Immunofluorescence staining of NF-κB P65 in tenocytes following 1 h of IL-1β, TNFα and IFN-γ stimulation with or without IL-1RA. DAPI staining of the nucleus is shown in blue. Images are representative of three biological replicates. Scale bar = 50 µm. Fold change in tendon-associated (**c**) and *MMP* (**d**) gene expression in tenocytes following stimulation with inflammatory cytokines and/or IL-1RA compared to the no cytokine control. Number sign indicates significant rescue effect (*p* < 0.05). Error bars represent the S.E.M of three biological replicates of tenocytes. Cells in these experiments were between P4 and P10

IL-1RA blocked the effect of IL-1β on 3-D collagen gel contraction by tenocytes (Fig. 7a) as published previously (McClellan et al. 2019a). However, IL-1RA was unable to rescue collagen gel contraction, or nuclear translocation of NF-κB (Fig. 7b–b’’’’), when IFN-γ, TNFα and IL-1β were used in combination. Gene expression analysis demonstrated IL-1RA alone had no significant effect on tendon-associated or *MMP* gene expression in tenocytes. Although IL-1RA can fully rescue IL-1β induced 3-D contraction by tenocytes, it did not fully rescue gene expression. While a trend for rescue was seen for most genes, this rescue effect was only significant for *SCX, MMP8* and *MMP13* (Fig. 7c and d). In comparison, IL-1RA demonstrated minimal impact in rescuing the combined effects of all three cytokines on tendon-associated gene expression. However, IL-1RA did appear to reduce the adverse effects a combination of all three cytokines has upon *MMP* expression, although this effect was not significant for any gene examined.

### Equine BM-MSCs are unable to protect tenocytes from the negative consequences of inflammatory cytokines

**Fig. 8:**
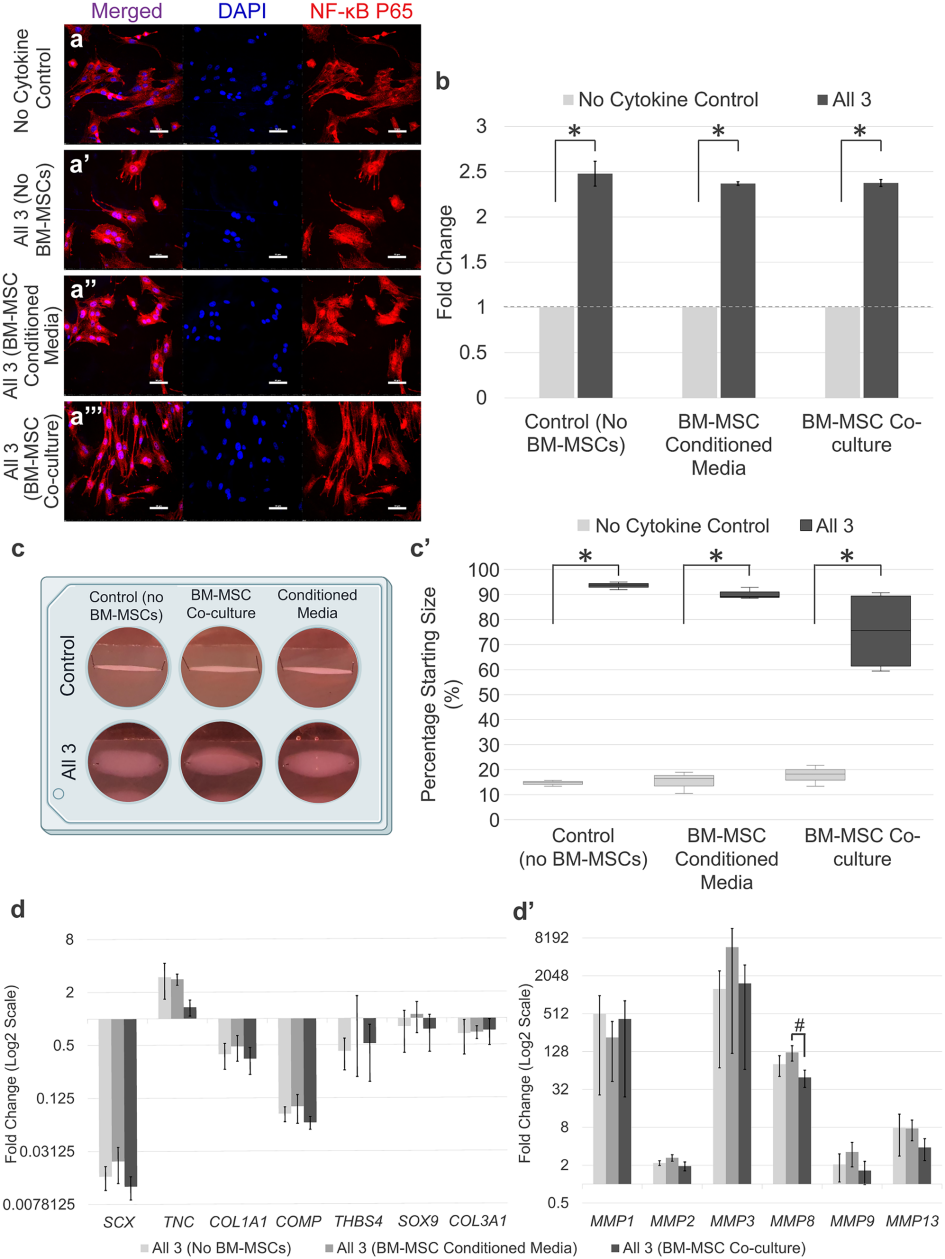
BM-MSCs are unable to release soluble factors which protect tenocytes from combined inflammatory stimulation. Immunofluorescence demonstrates neither BM-MSC-conditioned media (**a**’’) nor BM-MSCs in co-culture (**a**’’’) are able to prevent the nuclear translocation of NF-κB P65 in tenocytes following cytokine stimulation (**a**–**a**’’’). DAPI staining of the nucleus is shown in blue. Images are representative of four biological replicates of tenocytes. Scale bar = 50 µm. (**b**) Quantification of the relative nuclear fluorescent intensity of NF-κB following cytokine stimulation. Data is shown as fold change compared to the no cytokine control. Error bars represent the S.E.M of four biological replicates of tenocytes. Asterisk indicates the fold change in nuclear fluorescent intensity is significantly different to the no cytokine control (*p* < 0.05). (**c**–**c**’) BM-MSC-conditioned media or BM-MSCs in co-culture were unable to rescue the adverse effects inflammatory cytokines have upon 3-D collagen gel contraction by tenocytes. Representative collagen gel images are shown from day 14 (**c**). Asterisk indicates the degree of collagen gel contraction is significantly different to the no cytokine control (right; *p* < 0.05). Fold change in tendon-associated (**d**) and *MMP* (**d**’) gene expression in tenocytes following inflammatory cytokine stimulation and culture with no BM-MSCs, BM-MSC-conditioned media or BM-MSCs in co-culture compared to the no cytokine control. Number sign indicates significant difference between the groups (*p* < 0.05). Error bars represent the S.E.M of four biological replicates of tenocytes. Cells in these experiments were between P3 and P10

Flow cytometry analysis showed the BM-MSCs were positive for CD90 (98.2%) and CD29 (95.4%), but negative for CD14 (0.82%) and CD45 (0.26%) (Fig. S6). Immunofluorescence demonstrated that neither BM-MSCs in transwell co-culture nor BM-MSC-conditioned media were able to prevent NF-κB P65 nuclear translocation in tenocytes stimulated with IFN-γ, TNFα and IL-1β in combinations compared to the no BM-MSC control (Fig. 8a–a’’’ and b). In 3-D culture, conditioned media taken from actively proliferating BM-MSCs failed to rescue collagen gel contraction by tenocytes following IFN-γ, TNFα and IL-1β stimulations (Fig. 8c–c’). When BM-MSCs were co-cultured with the 3-D cultures of tenocytes, there was a small rescue effect; however, this was highly variable, and the 3-D collagen gels remained significantly different to the no cytokine control. Furthermore, there was no significant difference in the final degree of 3-D collagen gel contraction by tenocytes between BM-MSC culture conditions. Crystal violet staining showed no difference in BM-MSC survival between conditions at day 14 of 3-D co-culture (Fig. S7). In accordance with the 3-D collagen gel contraction data, neither BM-MSC-conditioned media nor BM-MSCs in co-culture were able to rescue adverse alterations in tendon-associated or *MMP* gene expression resulting from IFN-γ, TNFα and IL-1β stimulations (Fig. 8d–d’).

## Discussion

Inflammation plays a key role in both normal and pathogenic tendon regeneration (Rees et al. 2014). However, our understanding of the inflammatory mechanisms underpinning inferior tendon healing is limited, meaning successful targeted therapeutics are lacking. Our previous work demonstrated IL-1β has adverse effects on equine tenocytes in vitro (McClellan et al. 2019a); however, during tendon injury in vivo, other pro-inflammatory cytokines are also present. Therefore, here we investigated the combined effects of IFN-γ, TNFα and IL-1β on inflammatory pathway activation and tendon cell properties.

Insights into the pathogenic effects of inflammatory cytokines may be obtained by studying specific transcription factors induced by those cytokines. The canonical NF-κB pathway is an important regulator of the inflammatory response, and inappropriate modulation of NF-κB has been implicated in the formation of fibrous tendon tissue (Abraham et al. 2019; Best et al. 2019). Activation of the NF-κB pathway results in the rapid translocation of the NF-κB P65 dimer from the cytoplasm to the nucleus where it modulates gene transcription. Previously, we demonstrated IL-1β stimulation induces the nuclear translocation of NF-κB P65 in equine tenocytes (McClellan et al. 2019a). Here, we demonstrate that NF-κB signalling is also activated by TNFα stimulation, but not by IFN-γ. This is in contrast to studies in HeLa S3 cells, where IFN-γ induced NF-κB DNA binding (Deb et al. 2001). This suggests that the direct activation of NF-κB by IFN-γ is restricted to specific cell lineages or types. In addition to NF-κB, the inflammatory pathways JNK, STAT1 and P38 MAPK were also examined due to their roles in activating genes involved in immune function (Gough et al. 2008; Wang et al. 2019). Despite IFN-γ, TNFα and IL-1β having little effect in the activation of JNK or P38 MAPK, IFN-γ did show upregulation of STAT1 signalling. This is in accordance with previous research, and the importance of STAT1 in IFN-γ biology has been demonstrated, as deficits in immunity in STAT1 knockout mice are notably similar to the phenotypes of IFN-γ knockout mice (Durbin et al. 1996). It is possible that other inflammatory pathways such as STAT3 and STAT5 are involved in the tendon healing response (Tarafder et al. 2017). Therefore, further research examining the phosphorylation profile of a broad array of inflammatory pathway proteins following inflammatory stimuli would be beneficial to identify additional pathways of interest.

Investigation of commonly cited tendon-associated genes revealed significant changes in expression levels following cytokine stimulation. Most notably, the expression of *SCX*, *COL1A1* and *COMP* was significantly downregulated following stimulation with TNFα and IL-1β. Although not significant, the greatest downregulation was observed when all three cytokines were used in combination, suggesting a synergistic effect. These observed changes in gene expression have previously been suggested to negatively impact tenocytes and their ability to regenerate a healthy tendon matrix (McClellan et al. 2019a). We also identified considerable increases in *MMP* gene expression in tenocytes following stimulation with TNFα and IL-1β. Metalloproteinases play an integral part in the maintenance and repair of the extracellular matrix (ECM), and evidence suggests alterations to the synthetic-degradative equilibrium of these enzymes underlies tendon degeneration (Jones et al. 2006; Robertson et al. 2012). It has been suggested that changes in *MMP* gene expression are directly related to NF-κB activation, with studies identifying *MMP1* (Vincenti et al. 1998), *MMP3* (Borghaei et al. 2004) and *MMP9* (Bond et al. 1998) as direct targets of NF-κB signalling. However, to the authors knowledge, it is unknown whether all the genes examined here are direct targets of NF-κB and further work to establish this is warranted.

The ability of tenocytes to contract 3-D collagen gels has widely been used to measure cell-mediated matrix reorganisation (Bell et al. 1979; Grinnell and Petroll 2010; Yang et al. 2015). Previously, we have demonstrated the degree of 3-D collagen gel contraction by tenocytes is significantly impaired by IL-1β (McClellan et al. 2019a). Our results demonstrate multi-cytokine stimulation with IL-1β, TNFα and IFN-γ significantly impairs 3-D collagen gel contraction by tenocytes to a greater extent than when TNFα or IL-1β are used individually. Taken together with the increased *MMP* gene expression evidenced following cytokine stimulation, these results suggest tenocytes in an inflammatory environment contribute to matrix degeneration rather than regeneration. However, MMP protein activity, tissue inhibitor of metalloproteinase (TIMP) expression, MMP:TIMP ratios and specific collagen type ratios remain to be measured to determine if this is the case.

Of note, our results revealed that IFN-γ, which did not activate NF-κB, had no significant effect on 3-D collagen gel contraction or *MMP* gene expression. We have previously demonstrated that IFN-γ results in increased expression of MHC I in tenocytes (McClellan et al. 2019b), and this may be mediated by the activation of STAT1 (Lieberman et al. 2004). Additionally, our results further indicate that IFN-γ can synergize with IL-1β and TNFα to cause a greater effect on 3-D collagen gel contraction than when the cytokines are used individually. Previous reports in a vascular endothelium cell line have found that although IFN-γ on its own does not significantly induce NF-κB, it potentiates TNFα-induced NF-κB nuclear translocation (Cheshire and Baldwin 1997). The mechanisms behind this synergistic effect may be due to a cooperative relationship between STAT1 and NF-κB, where certain genes are only expressed when both transcription factors are activated simultaneously (Hiroi and Ohmori 2005).

The secretion of interleukin 6 (IL-6) was significantly upregulated by tenocytes following stimulation with IL-1β and a combination of all three cytokines. Interestingly, IL-6 secretion has been well documented as a downstream effector of NF-κB activation and is elevated during tendon ruptures (Jové et al. 2005; Novotny et al. 2008; Millar et al. 2016; Liu et al. 2017). Nevertheless, the action of IL-6 in tendinopathy is complex. IL-6 is considered as a classical pro-inflammatory cytokine due to its role in macrophage activation (Stolk et al. 2017). However, it is also known to possess anti-inflammatory properties by promoting the secretion of factors such as IL-10 and IL-1RA (Lin et al. 2006). Our results indicate that IL-6 stimulation alone has little effect on tendon-associated gene expression and 3-D collagen gel contraction by tenocytes. Interestingly, like IFN-γ, IL-6 also failed to activate NF-κB signalling. Overall, this suggests IL-6 has limited catabolic effects on the parameters investigated in this in vitro study. This finding is similar to previous research in tenocytes (John et al. 2010), which, however, is in stark contrast to work conducted in bovine articular chondrocytes demonstrating IL-6 upregulates the expression of *MMPs* (Legendre et al. 2005). Nevertheless, the expression of IL-6 has been shown to be imperative for normal tendon healing (Lin et al. 2006); therefore, it is clear that more detailed analyses into its immunoregulatory role are needed.

Our study also evaluated whether currently used inflammatory targeting treatments were able to protect tenocytes from a multi-cytokine environment in vitro. IL-1RA is a naturally occurring inhibitor of IL-1β and functions by competing with IL-1β for binding to the IL-1 receptor 1 (Fredberg and Ostgaard 2009). IL-1RA-based therapies, such as Anakinra, have been advocated for the treatment of inflammatory disorders such as rheumatoid arthritis (Bresnihan et al. 1998; Hallegua and Weisman 2002; Dragoljevic et al. 2020). Since IL-1β is implicated in tendinopathy (Hosaka et al. 2002; Morita et al. 2017), IL-1RA may have therapeutic potential to improve tendon regeneration (Geburek et al. 2015). Nonetheless, anakinra injection in human patients with chronic Achilles tendinopathy demonstrated little beneficial effect, with no significant reduction seen in pain or intratendinous blood flow, and increased tendon thickness after 12 weeks of treatment (Fredberg and Ostgaard 2009). Our results do suggest some beneficial effects of IL-1RA, but only when IL-1β was used alone. Treatment with IL-1RA was able to fully rescue the adverse effects of IL-1β on 3-D collagen gel contraction by tenocytes but was unable to fully rescue tendon-associated gene expression. This suggests that we were unable to completely block IL-1β signalling, and based on the previous literature, it is possible a higher concentration of IL-1RA may have been needed (Hallegua and Weisman 2002). Nevertheless, our work demonstrates that IL-1RA cannot prevent the adverse effects of multiple cytokine stimulation on tenocytes. Since the primary goal of IL-1RA is to inhibit the inflammatory cascade induced by IL-1β, instead of directly aiding tissue regeneration, future treatment options that directly target the inflammatory cascades activated by both IL-1β and TNFα may be a more effective treatment option.

It is well established that BM-MSCs possess both anti-inflammatory and immunosuppressive properties (Aggarwal and Pittenger 2005; Zhang et al. 2017). However, the detailed mechanisms by which BM-MSCs modulate inflammation to improve tendon regeneration remain unclear. Potential theories include the following: BM-MSCs modulate the activity of macrophages (Kim and Hematti 2009; Manning et al. 2015), BM-MSCs secrete factors which modulate tenocyte activity (van Buul et al. 2012) or that BM-MSCs inactivate pro-inflammatory cytokines directly (Viganò et al. 2019). Using a BM-MSC/tenocyte co-culture model, we demonstrated that BM-MSCs were unable to directly modulate tenocyte activity and protect these cells from inflammatory stimuli. This result is supported by previous studies in mice where MSCs derived from adipose tissue (ASCs) were unable to protect tendon fibroblasts from IL-1β stimulation (Manning et al. 2015). Although the in vitro methods used here provide a well-controlled environment to determine tenocyte responses to specific cytokine combinations and BM-MSC interventions, it does not fully reproduce the complexities of tendinopathy in vivo where multiple cell types, including immune cells, are present and cells have direct cell-to-cell contact. Manning et al. (2015) found that ASCs suppressed the negative effects of M1 (pro-inflammatory) macrophages on tendon fibroblasts by inducing a phenotypic switch to an M2 (anti-inflammatory) macrophage. Therefore, our results are more consistent with BM-MSCs exhibiting their beneficial effects via circulating macrophages rather than the tenocytes themselves. Consequently, immune cell manipulation towards a regenerative phenotype may provide a promising therapeutic option for superior tendon healing.

Under pro-inflammatory conditions, BM-MSCs become “primed” and upregulate major histocompatibility complex (MHC) expression in order to enhance their immunomodulatory properties (Noronha et al. 2019). Here, we did not “prime” the BM-MSCs prior to use, and the conditioned media came from BM-MSCs that had not been exposed to inflammatory cytokines therefore this may have affected our results. However, the BM-MSCs used in co-culture with the tenocytes were in direct contact with IFN-γ, TNFα and IL-1β throughout the experiments. IFN-γ in particular is identified as one of the most potent activators of BM-MSCs immunomodulatory properties (Krampera et al. 2006; De Witte et al. 2016); therefore, we do not afford the lack of “priming” to the lack of protective effects afforded to tenocytes here. Furthermore, we also performed the work using BM-MSC-conditioned media in 10% serum. This was because the tenocytes are unable to contract a 3-D collagen gel over a 2-week period in low serum concentrations. However, the high serum levels we used may have masked the effects of components produced by the BM-MSCs.

There are other limitations to our study. For instance, our gene expression experiments were performed using cells cultured in 2-D monolayer to enable us to make direct comparisons to our previous work on IL-1β (McClellan et al. 2019a). However, we have recently reported that the distinct gene expression profiles of tenocytes are better preserved in a 3-D culture environment (Paterson et al. 2020) and future work in 3-D may enable the capture of differences between experimental groups that may be lost in conventional 2-D culture systems. Additionally, our gene expression experiments were performed using cells up to passage 10, and it has been suggested that cells may change their gene expression profiles with increasing passage. However, both ourselves and other researchers have demonstrated that although changes in tendon-associated gene expression are found to occur between P0 and some of the very early passages, they then remain stable up to passage 10, therefore, we feel our results do provide an accurate in vitro model (Jo et al. 2019; Liao et al. 2020; Paterson et al. 2020). Furthermore, here we added the inflammatory stimulus at the same time as the protective therapy (IL-1RA or BM-MSCs), whereas in clinical cases, this would not be the case. This approach was chosen because the 3-D cultures can only be maintained for 2 weeks (Barsby et al. 2014) and we wanted to provide the maximum opportunity for the protective therapy to have an effect. Future studies aiming to identify novel therapies for tendinopathy should consider adding the therapy subsequently to inflammation. Finally, the inflammatory cytokine concentrations used here are based on previous in vitro studies of a similar type. However, it is unknown if these concentrations correlate to the in vivo environment due to a lack of investigation of cytokine concentrations in equine tendon tissue (Ellis et al. 2022).

In conclusion, we have shown that TNFα and IL-1β both activate NF-κB signalling and together induce negative effects in tenocytes which cannot be rescued by conventional therapies such as IL-1RA or BM-MSCs. A superior approach for treatment of tendinopathy may therefore involve the targeting of specific, shared signalling pathways such as NF-κB.

## Supplementary Information

Below is the link to the electronic supplementary material.

Supplementary file1 (DOCX 4886 KB)

## Data Availability

All relevant data are within the manuscript and its supplementary information files.
