# Supplementary material for: Tumour necrosis factor alpha, interleukin 1 beta and interferon gamma have detrimental effects on equine tenocytes that cannot be rescued by IL-1RA or mesenchymal stromal cell–derived factors
Source: Cell Tissue Res. 2022 Dec 22;391(3):523–44. doi: 10.1007/s00441-022-03726-6 (PMC9974687; doi:10.1007/s00441-022-03726-6)
Supplement: Supplementary file 1 — Supplementary file1 (DOCX 4886 KB) [file 441_2022_3726_MOESM1_ESM.docx]

**Cell and Tissue Research**

**Tumour necrosis factor alpha, Interleukin 1 beta and Interferon gamma have detrimental effects on equine tenocytes that cannot be rescued by IL-1RA or mesenchymal stromal cell-derived factors**

**Emily J. Smith^1*^**, Ross E. Beaumont^1^, Alyce McClellan^2^, Cheryl Sze^2^, Esther Palomino Lago^1^, Liberty Hazelgrove^1,3^, Jayesh Dudhia^1^, Roger K. W. Smith^1^, Deborah J. Guest^1*^

^1^Department of Clinical Sciences and Services, The Royal Veterinary College, Hawkshead Lane, North Mymms, Hatfield, Herts, AL9 7TA, UK.

^2^Centre for Preventative Medicine, Animal Health Trust, Newmarket, Suffolk, CB8 7UU, UK.

^3^Kingston University, River House, 53-57 High Street, Kingston upon Thames, Surrey, KT1 1LQ, UK.

*Correspondence and requests for materials should be addressed to Deborah J. Guest (email: [djguest@rvc.ac.uk](mailto:djguest@rvc.ac.uk)) or Emily J. Smith (email: [ejsmith@rvc.ac.uk](mailto:ejsmith@rvc.ac.uk))

**
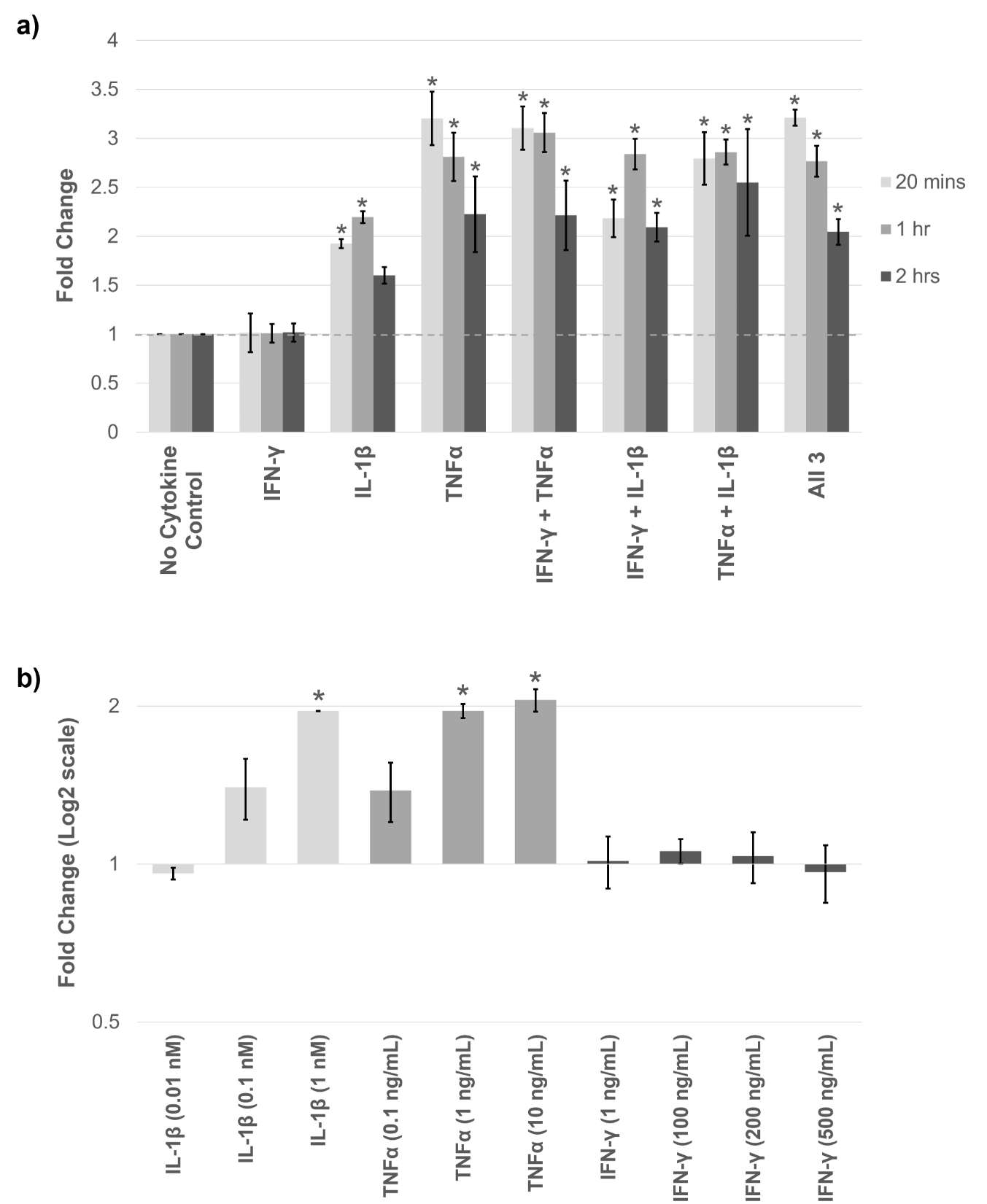
Supplementary Figures**

**Fig. S1** Time and dose response experiments to establish optimal inflammatory cytokine conditions in adult tenocytes. (a) Quantification of the relative nuclear fluorescent intensity of NF-κB P65 immunofluorescence following 20 minutes, 1 hr and 2 hrs of cytokine stimulation. (b) Quantification of the relative nuclear fluorescent intensity of NF-κB P65 immunofluorescence following stimulation with various concentrations of the inflammatory cytokines for 20 minutes. All data shown as fold change compared to the no cytokine control. Error bars represent the S.E.M of three measurements from each of three biological replicates. * Indicates the fold change in nuclear fluorescent intensity is significantly different to the no cytokine control (p<0.05). Cells in these experiments were between P3 and P10


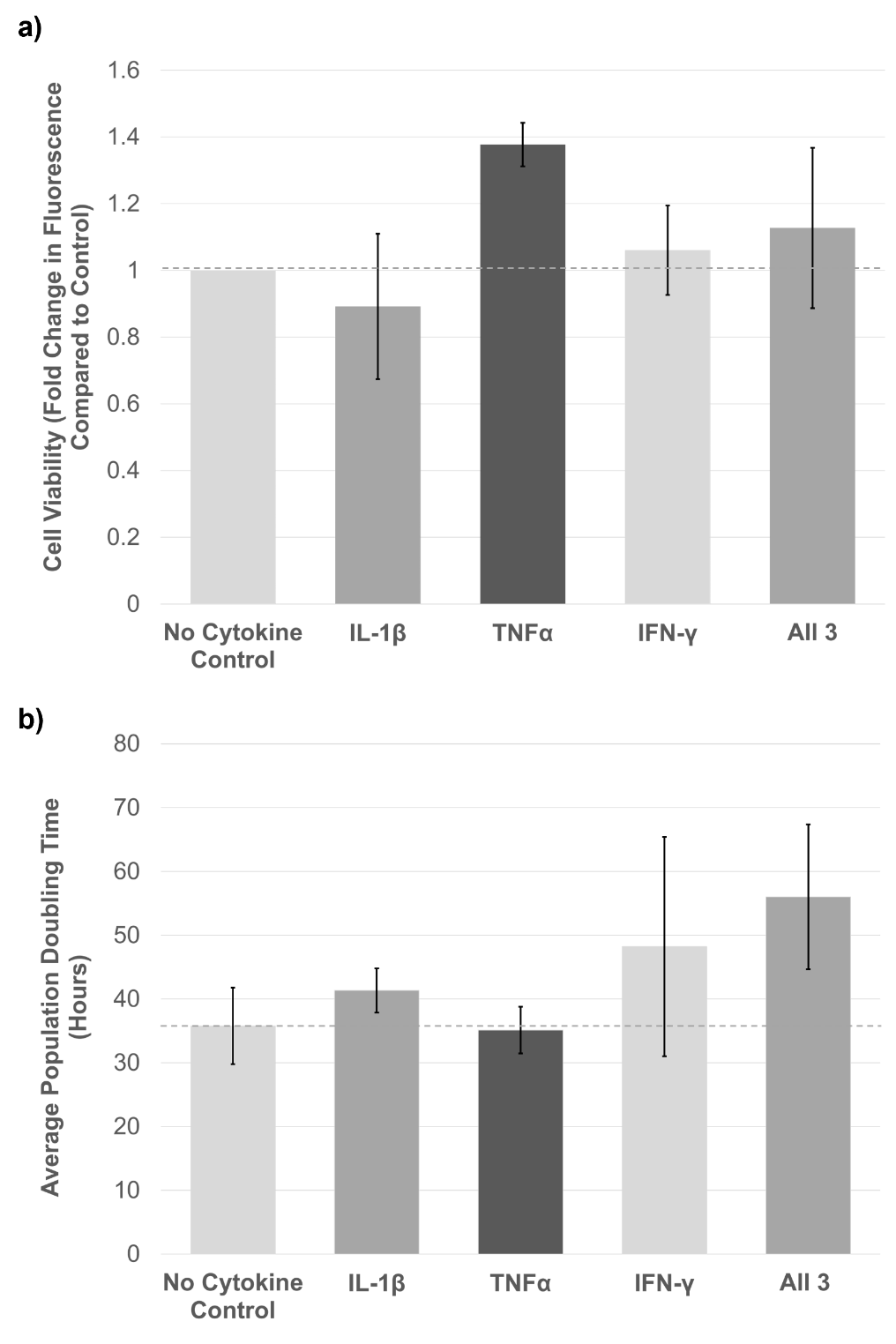
**Fig. S2** Effects of inflammatory cytokines on cell proliferation and viability in tenocytes. **(a)** Presto blue assay showing tenocytes stimulated with IFN-γ, TNFα and IL-1β for 72 hrs showed no significant effect in cell viability compared to the no cytokine control at P<0.05. **(b)** Inflammatory cytokine stimulation for 72 hrs has no significant effect on population doubling time of tenocytes. All experiments were performed using three biological replicates of tenocytes. Error bars represent the S.E.M of three biological replicates of tenocytes. Cells in these experiments were used between P4 and P11


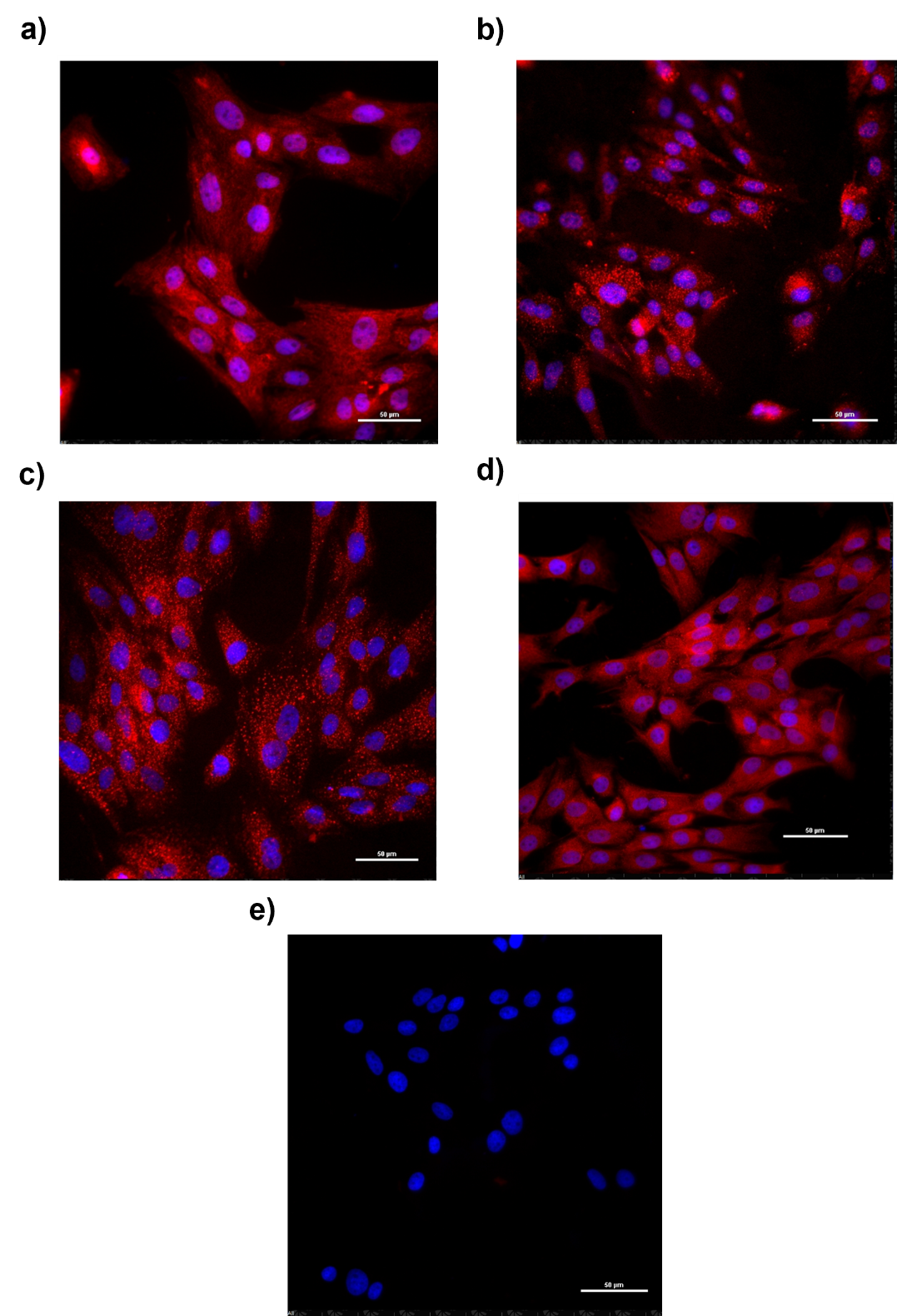


**Fig. S3** Expression of TNFα and IFN-γ receptors in tenocytes. Immunofluorescent staining of the TNFα signalling receptors TNFR1 **(a)** and TNFR2 **(b),** and the IFN-γ signalling receptors IFNGR1 **(c)** and IFNGR2 **(d)** in unstimulated tenocytes (red staining). Secondary antibody only control shown in **(e)**. DAPI staining of the nucleus is shown in blue. Scale bar = 50 μm. Images are representative of three biological replicates. Cells in these experiments were used between P6 and P7


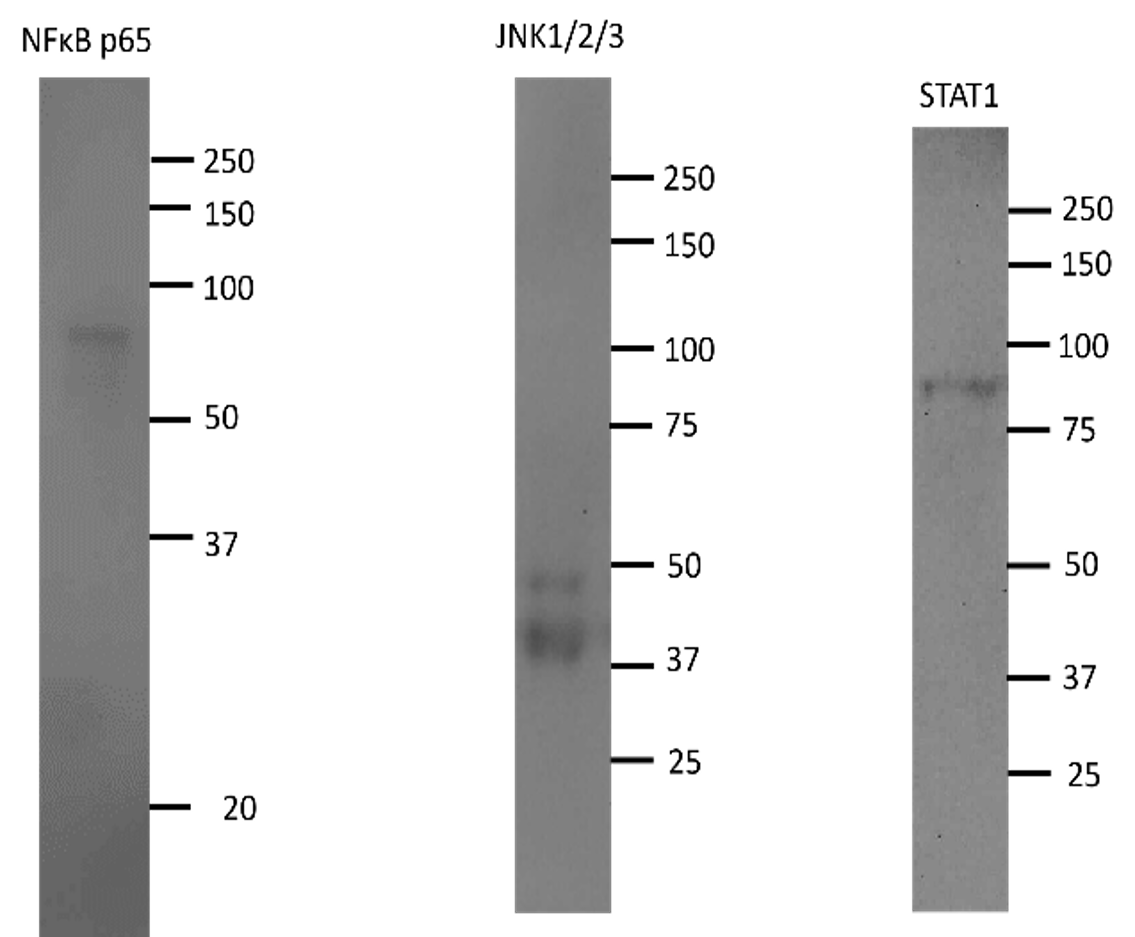


**Fig. S4** Western blot of primary antibodies. Western blotting showing binding of NF-κB p65, JNK and STAT1 antibodies to equine whole cell protein from skin fibroblasts


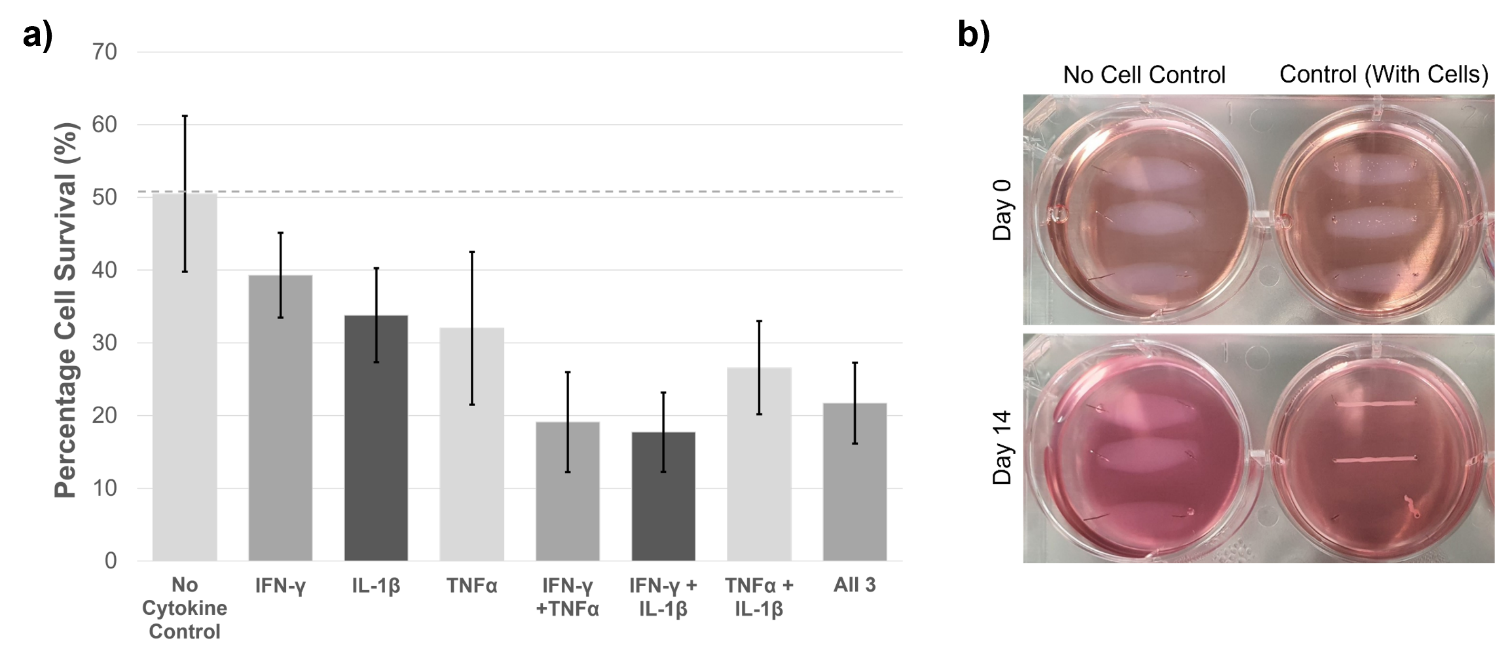


**Fig. S5** Equine tenocyte viability in 3-D collagen gels at day 14. (a) Percentage cell survival of tenocytes cultured in 3-D collagen constructs with inflammatory cytokine stimulation at day 14. Error bars represent the S.E.M of three biological replicates of tenocytes. Statistical analysis shows no significant difference between inflammatory cytokine conditions (*p =* 0.069). (b) 3-D collagen constructs lacking tenocytes fail to contract over 14 days. Representative images are shown. Cells in these experiments were between P4 and P10


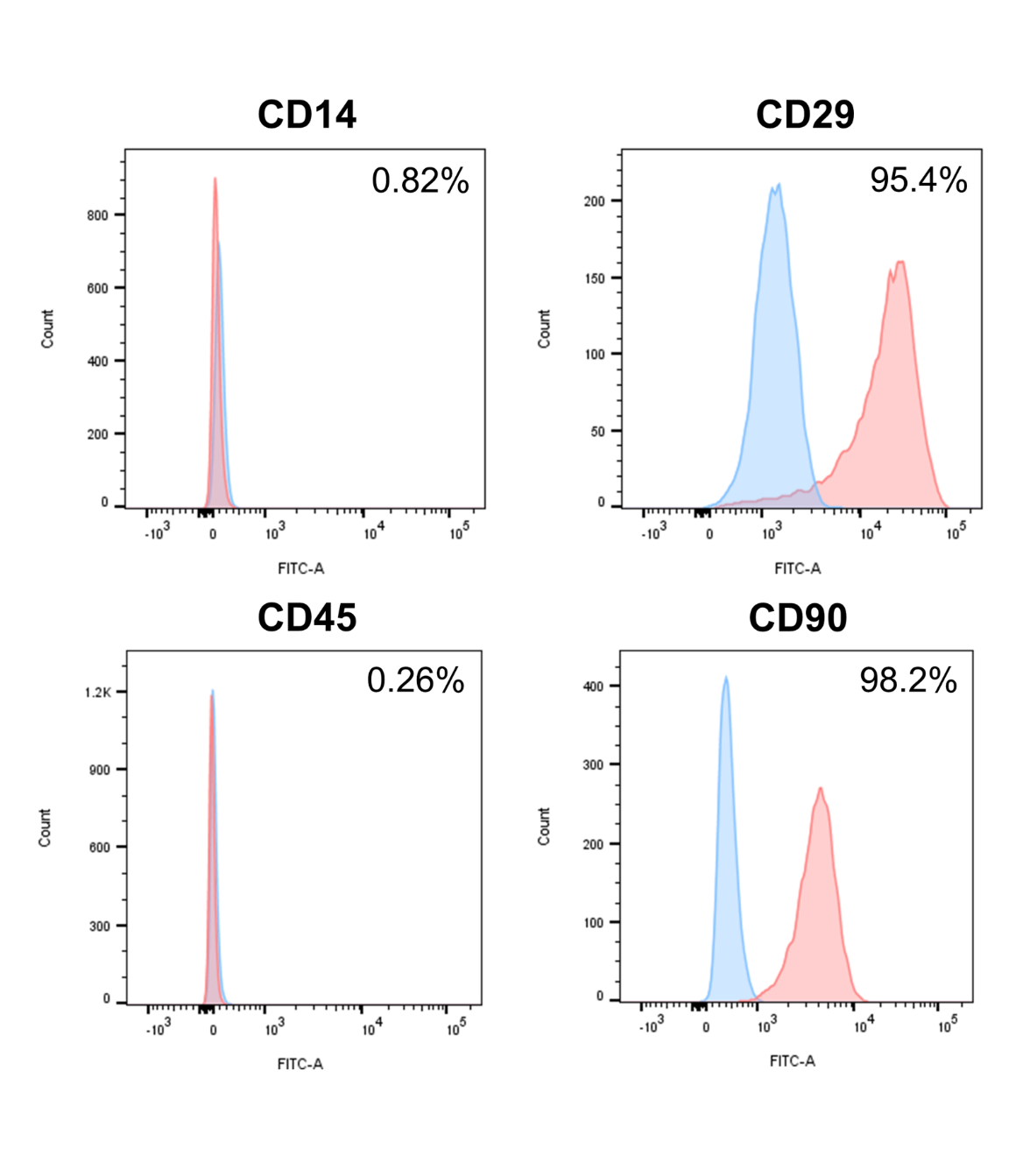


**Fig. S6** Immunophenotyping of equine BM-MSCs looking at cluster of differentiation markers. BM-MSCs showed positively for antibodies against CD90 and CD29, and negatively for CD45 and CD14. Representative histograms illustrate relative number of cells compared to the mean fluorescent intensity based. The blue histograms represent the relevant isotype control and the red histogram represents the marker antibody staining. Mean percentage of positive cells based on two biological replicates is also shown. Cells were used between P5 and P7


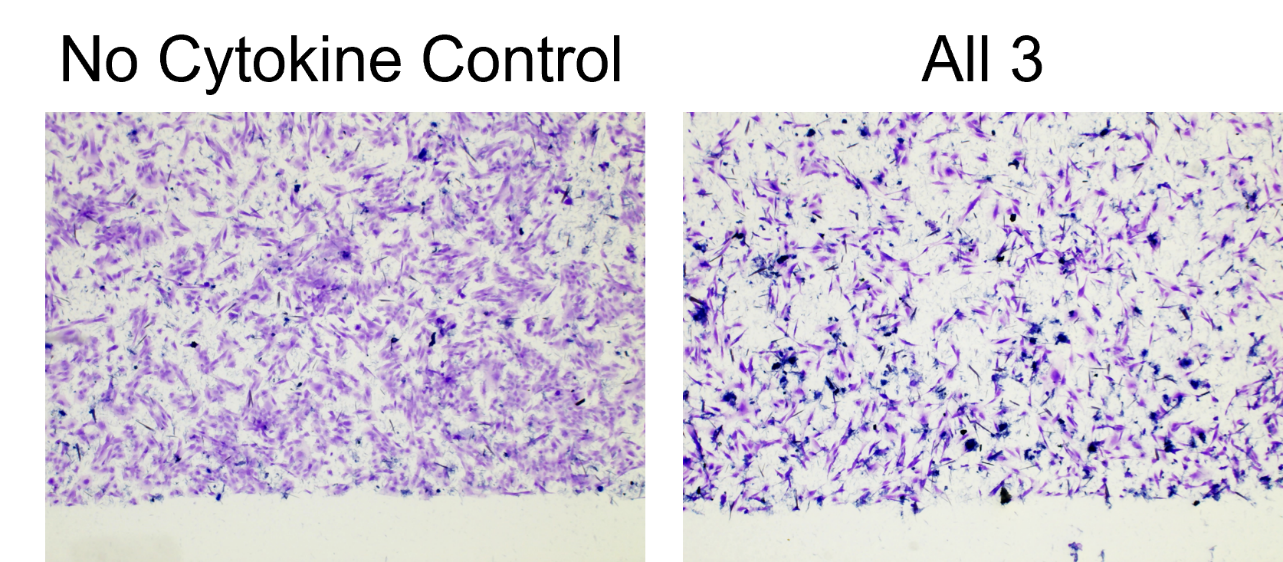


**Fig. S7** Crystal violet staining shows BM-MSC survival at day 14 of 3-D collagen gel contraction. Crystal violet staining of fixed BM-MSCs (no cytokine control and IL-1β, TNFα and IFN-γ stimulated) in 3-D tenocyte/BM-MSC co-culture at day 14. Images representative of three biological replicates
